# Diagnostic accuracy of alternative biomarkers for acute aortic syndrome: a systematic review

**DOI:** 10.1136/emermed-2023-213772

**Published:** 2024-08-06

**Authors:** Joshua Wren, Steve Goodacre, Abdullah Pandor, Munira Essat, Mark Clowes, Graham Cooper, Robert Hinchliffe, Matthew J Reed, Steven Thomas, Sarah Wilson

**Affiliations:** 1 Sheffield Teaching Hospitals NHS Foundation Trust, Sheffield, UK; 2 University of Sheffield, Sheffield, UK; 3 Aortic Dissection Charitable Trust, Sheffield, UK; 4 Department of Vascular Surgery, North Bristol NHS Trust, Westbury on Trym, UK; 5 Translational Health Sciences, University of Bristol, Bristol, UK; 6 Emergency Medicine Research Group Edinburgh (EMERGE), NHS Lothian, Edinburgh, UK; 7 Acute Care Group, The University of Edinburgh Usher Institute of Population Health Sciences and Informatics, Edinburgh, UK; 8 Emergency Department, Wexham Park Hospital, Slough, UK

**Keywords:** systematic review, arterial, cardiovascular system

## Abstract

**ABSTRACT:**

**Background:**

D-dimer is the only biomarker currently recommended in guidelines for the diagnosis of acute aortic syndrome (AAS). We undertook a systematic review to determine whether any alternative biomarkers could be useful in AAS diagnosis.

**Methods:**

We searched electronic databases (including MEDLINE, EMBASE and the Cochrane Library) from inception to February 2024. Diagnostic studies were eligible if they examined biomarkers other than D-dimer for diagnosing AAS compared with a reference standard test in people presenting to the ED with symptoms of AAS. Case-control studies were identified but excluded due to high risk of bias. Selection of studies, data extraction and risk of bias assessments using the Quality Assessment of Diagnostic Accuracy Studies 2 (QUADAS-2) tool were undertaken independently by at least two reviewers. We used narrative synthesis to summarise the findings.

**Results:**

We identified 2017 citations, included 13 cohort studies (n=76–999), and excluded 38 case-control studies. Methodological quality was variable, with most included studies having unclear or high risk of bias and applicability concerns in at least one item of the QUADAS‐2 tool. Only two studies reported biomarkers with sensitivity and specificity comparable to D-dimer (ie, >90% and >50%, respectively). Wang *et al* reported 99.1% sensitivity and 84.9% specificity for soluble ST2; however, these findings conflicted with estimates of 58% sensitivity and 70.8% specificity reported in another study. Chun and Siu reported 95.6% sensitivity and 56.1% specificity for neutrophil count, but this has not been confirmed elsewhere.

**Conclusion:**

There are many potential alternative biomarkers for AAS but few have been evaluated in more than one study, study designs are often weak and reported biomarker accuracy is modest or inconsistent between studies. Alternative biomarkers to D-dimer are not ready for routine clinical use.

**PROSPERO registration number:**

CRD42022252121.https://www.crd.york.ac.uk/prospero/display_record.php?ID=CRD42022252121

**WHAT IS ALREADY KNOWN ON THIS TOPIC**

- D-dimer has some diagnostic value in the assessment of suspected acute aortic syndrome (AAS), but the role of other potential biomarkers is unclear.

**WHAT THIS STUDY ADDS**

- Our systematic review showed that the evidence for other biomarkers is weak and estimates of accuracy are generally modest or inconsistent.

**HOW THIS STUDY MIGHT AFFECT RESEARCH, PRACTICE OR POLICY**

- Diagnostic biomarkers for AAS, other than D-dimer, are not ready for routine clinical use.
- Large cohort studies are required to evaluate multiple biomarkers in an appropriate population with suspected AAS.

## Introduction

 Acute aortic syndrome (AAS) is a life-threatening emergency condition affecting the thoracic aorta that includes acute aortic dissection (AAD), intramural haematoma and penetrating ulcer. CT angiography (CTA) scanning of the aorta has high sensitivity and specificity for diagnosing AAS but incurs significant costs and the risks of ionising radiation.

Biomarkers could be used to select patients with suspected AAS for CTA. The pathophysiology of AAS allows researchers to select and investigate various biomarkers. In aortic dissection, intima rupture allows circulating blood to enter the media of the aorta, forming both true and false lumens. This results in an initial inflammatory response, followed by infiltration of inflammatory cells and subsequent vascular smooth muscle cell apoptosis, ultimately leading to aortic media degradation. This process potentiates aortic dilatation, aneurysm formation, progression to dissection and can lead to rupture.^1^ Recognising this condition early is paramount to avoid significant morbidity and mortality. Biomarkers can therefore be divided according to the process with which they are associated: clotting, inflammatory response, lipid metabolism, cardiac myocyte damage, vascular extracellular matrix damage and other protein metabolism.^2^ Biomarkers may also reflect the consequent effects of organ hypoperfusion. Such biomarkers would therefore play more of a role in severity of sequelae as opposed to identification of AAS.

D-dimer is the most extensively studied biomarker for AAS. The most recent meta-analysis included 18 studies with 7978 patients and reported pooled sensitivity of 96.5% (95% credible interval (CrI) 94.8% to 98%) and specificity of 56.2% (95% CrI 48.3% to 63.9%) for D-dimer above the diagnostic threshold of 500 ng/mL.^3 4^ D-dimer sensitivity can be improved by using it alongside a clinical probability score, such as the aortic dissection detection risk score (ADD-RS), which uses clinical features to estimate clinical risk on a score from 0 to 3. The combination of ADD-RS ≥1 and D-dimer >500 ng/mL in a recent meta-analysis of six studies demonstrated pooled sensitivity of 93.1% (95% CrI 87.1% to 96.3%) and specificity 67.1% (95% CrI 54.4% to 77.7%) when diagnosing AAS.^5^ These findings suggest a potential role for D-dimer, alone or alongside clinical probability scoring, to select patients for CTA, and suggest that alternative biomarkers with superior accuracy to D-dimer could have an important role in clinical practice.

A number of studies have evaluated biomarkers for AAS other than D-dimer. However, these have not yet been systematically examined to identify the most promising candidates for future research or to determine whether any have the potential to improve on the accuracy of D-dimer. We aimed to systematically review biomarkers other than D-dimer to determine their accuracy for diagnosing AAS.

## Methods

A systematic review was undertaken in accordance with the general principles recommended in the Preferred Reporting Items for Systematic Reviews and Meta-Analyses statement.^6 7^ This review was part of a larger National Institute for Health and Care Research-funded (January to December 2023) Aortic Syndrome Evidence Synthesis (ASES) project on diagnostic strategies for suspected AAS and was registered on the International Prospective Register of Systematic Reviews database (CRD42022252121).^8^

### Eligibility criteria

Prospective or retrospective studies reporting diagnostic accuracy metrics were eligible if they examined any biomarkers (other than D-dimer) for diagnosing AAS compared with a reference standard test (eg, a definitive imaging modality such as CTA, ECG-gated CTA, echocardiography, and magnetic resonance angiography or confirmed/excluded by operation and autopsy). The study population of interest in our review consisted of people (any age) presenting to the ED with symptoms of AAS, including those with new-onset chest, back or abdominal pain, syncope or symptoms related to perfusion deficit. Studies including people with AAS following major trauma or as incidental findings were excluded. We initially planned to include all study designs in the review but only include cohort studies in any meta-analysis. After undertaking initial searches, we amended the protocol to exclude case-control designs from the review due to the potential for high bias resulting in inaccurate estimates and lack of representativeness of test accuracy in a clinical setting.^9 10^

### Data sources and searches

Potentially relevant studies were identified through searches of several electronic databases including MEDLINE (OvidSP from 1946 to February 2024), EMBASE (OvidSP from 1974 to February 2024), and the Cochrane Library (https://www.cochranelibrary.com from inception to February 2024) by an experienced information specialist (MC), who is a member of the research team. The search strategy used free text and thesaurus terms and combined synonyms relating to the topic of interest (eg, AAS and diagnostic strategies) with diagnostic testing terms (adapted Scottish Intercollegiate Guidelines Network filter for identifying diagnostic studies). Searches were supplemented by hand-searching the reference lists of all relevant studies (including existing systematic reviews); forward citation searching of relevant articles; contacting key experts in the field and undertaking targeted searches of the World Wide Web using the Google search engine. No date or language restrictions were applied on any database. Further details on the search strategy can be found in online supplemental appendix S1.

### Study selection

All titles were examined for inclusion by one reviewer (ME) and any citations that clearly did not meet the inclusion criteria (eg, non-human, unrelated to AAS) were excluded. All abstracts and full-text articles were then examined independently by two reviewers (ME and AP). Any disagreements in the selection process were resolved through discussion or if necessary, arbitration by a third reviewer (SG) and included by consensus.

### Data extraction and quality assessment

Data relating to study design, methodological quality and outcomes were extracted by one reviewer (JW) into a standardised data extraction form and independently checked for accuracy by a second (AP). Any discrepancies were resolved through discussion to achieve agreement. Where differences were unresolved, a third reviewer’s opinion was sought (SG). Where multiple publications of the same study were identified, data were extracted and reported as a single study.

The methodological quality of each included study was assessed using the Quality Assessment of Diagnostic Accuracy Studies-2 (QUADAS-2) tool.^11^ This instrument evaluates four key domains: patient selection, index test, reference standard, flow and timing. Each domain is assessed in terms of risk of bias and concerns regarding the applicability of the study results (first three domains only). The subdomains about risk of bias include a number of signaling questions to help guide the overall judgement about whether a study is at high, low or an unclear (in the event of insufficient data in the publication to answer the corresponding question) risk of bias.

### Data synthesis and analysis

We were unable to perform meta-analysis due to the limited number of studies per biomarker and variable reporting of items. As a result, a narrative synthesis approach was undertaken, with data being summarised in tables with accompanying narrative summaries that included a description of the included variables, statistical methods and performance measures (eg, sensitivity, specificity).^12 13^ All analyses were conducted using Microsoft Excel 2010 (Microsoft, Redmond, Washington, USA).

### Patient and public involvement

Two members of the Aortic Dissection Charitable Trust (https://aorticdissectioncharitabletrust.org/) joined the ASES project management team and helped to develop the study proposal. SG presented the findings of this review to a webinar of Aortic Dissection Charitable Trust members and sought their feedback on interpretation of the results.

## Results

### Study flow

**Figure 1:**
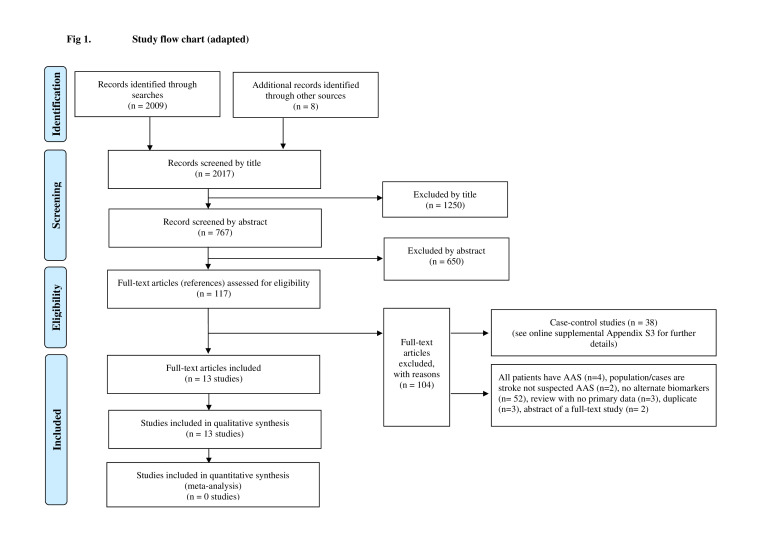
Study flow chart (adapted with permission from reference ^7^). AAS, acute aortic syndrome.

Figure 1 summarises the process of identifying and selecting relevant literature. Of the 2017 citations identified, 13 studies investigating 17 index tests met the inclusion criteria.^14^,^26^ The majority of the articles were excluded primarily on the basis of an inappropriate target population (patients with AAS or not suspected AAS), investigating intervention not an alternative biomarker or an unsuitable publication type (ie, reviews or abstract of full-text studies). A full list of excluded studies with reasons for exclusion can be found in online supplemental appendix S2. More specifically, 38 case-control studies were excluded due to the high potential for bias with this design.^9 10^ Four case-control studies reported comparisons with unselected controls with suspected AAS,^27^,^29^ whereas 34 case-control studies reported comparisons with healthy controls or controls with other diagnoses.^30^,^63^ A summary of the design and patient characteristics of the 38 excluded case-control studies can be found in online supplemental appendix S3. These studies evaluated a wide variety of biomarkers using a variety of different control groups. These studies may identify biomarkers for future research but do not provide reliable estimates of accuracy to inform clinical practice.

### Study and patient characteristics

The design and patient characteristics of the 13 included studies are summarised in table 1. Sample size ranged from n=76–999, with prevalence of AAS ranging from 1% to 51.2%. A team of researchers in Italy undertook five of the studies, evaluating the following biomarkers in consecutive cohorts: matrix metalloproteinases (MMP) 8 and 9, lactate dehydrogenase (LDH), white blood cell (WBC) count, platelet count, fibrinogen, copeptin and soluble suppression of tumourigenicity-2 (sST2). The other studies were undertaken in China (five studies), Germany, Japan and Canada, evaluating the following biomarkers: troponin, α-smooth muscle actin (α-SMA), smooth muscle myosin heavy chain (smMHC), soluble elastin fragments (sELAF) in serum, polycystin-1 (PC1), acidic and basic calponin at 6 and 24 hours after presentation, sST2, neutrophil-to-lymphocyte ratio, neutrophil count and leucocyte count.

**Table 1:** Study characteristics of the 13 included cohort studies

Study	Country	Cohort(n with AAS/N in cohort)	Selection process	Biomarkers evaluated	Method of measurement	Reference standard
Chun and Siu^14^	China	198/534	Consecutive patients	Neutrophil count	Automated analyser	CTA
Giachino *et al*^15^	Italy	52/126	Consecutive patients	Plasma MMP-8 and MMP-9	ELISA	Chest and abdominal CT with contrast
Lian *et al*^16^	China	49/179	Consecutive patients	Acidic calponin	ELISA	CTA
Meng *et al*^17^	Canada	2/201	Convenience sampling	Troponin	NR	CTA
Morello *et al*^18^	Italy	201/999	Convenience sampling	LDH	Plasma LDH assay	CTA, TEE
Morello *et al*^19^	Italy	110/891	Consecutive patients	WBC count, platelet count and fibrinogen	Assayed with automatic counter	CTA
Morello *et al*^20^	Italy	104/313	Convenience sampling	Copeptin	BRAHMS KRYPTOR automated method	CTA; if unavailable, 14-day clinical follow-up
Morello *et al*^21^	Italy	88/297	Not reported	sST2	ELISA	CTA, TEE; if unavailable, 30-day clinical follow-up
Peng *et al*^22^	China	35/76	Not reported	α-SMA, smMHC, sELAF in serum, PC1	ELISA	CTA
Suzuki *et al*^23^	Japan	59/217	Convenience sampling	Calponin (acidic and basic)	Sandwich-type enzyme immunoassay	Confirmed on imaging (type not specified)
von Kodolitsch *et al*^24^	Germany	128/250	Consecutive patients	Leucocyte count	NR	CTA, MRI, TEE, digital angiography, autopsy
Wang *et al*^25^	China	114/333	Not reported	sST2 and troponin	sST2—ELISA; troponin—NR	CT
Zhang *et al*^26^	China	323/697	Consecutive patients	Neutrophil-to-lymphocyte ratio	Automated haematology analyser	Blinded clinical review of imaging

CTA, CT angiography; LDH, lactate dehydrogenase; MMP, matrix metalloproteinase; NR, not reportedPC1, polycystin-1; sELAF, soluble elastin fragments; smMHC, smooth muscle myosin heavy chain; sST2, soluble suppression of tumourigenicity-2; TEE, trans-oesophageal echocardiography; WBC, white blood cellα-SMA, α-smooth muscle actin

### Risk of bias and applicability assessment

**Figure 2:**
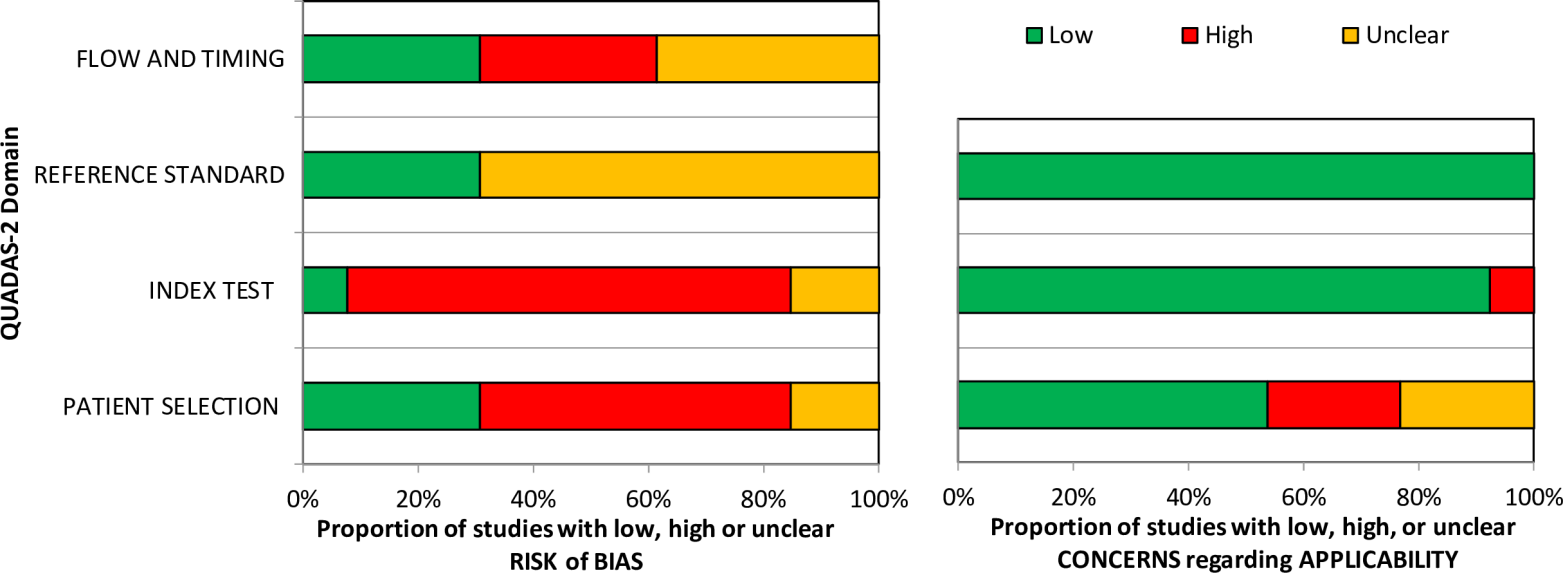
Quality Assessment of Diagnostic Accuracy Studies 2 (QUADAS-2) assessment summary graph—review authors’ judgements.

The overall methodological quality of the 13 included studies is summarised in table 2 and figure 2^1517^,^26^ and in the online supplemental appendix S4. The methodological quality of the included studies was variable, with most studies having unclear or high risk of bias and applicability concerns in at least one item of the QUADAS‐2 tool. The following sections attributed a high level of bias: patient selection, primarily due to the use of convenience sampling; index test, due to the absence of prespecified threshold values and flow and timing, principally because patients received different reference standards (imaging or follow-up). The reference standard item attributed an unclear level of bias due to a lack of clarity as to whether the reference standard results were interpreted without knowledge of the index test.

**Table 2:** QUADAS-2 quality assessment summary—review authors’ judgements

Study	Risk of bias				Applicability concerns		
	Patient selection	Index test	Reference standard	Flow and timing	Patient selection	Index test	Reference standard
Chun and Siu^14^	High	Unclear	Unclear	Unclear	Low	Low	Low
Giachino *et al*^15^	Low	High	Unclear	Unclear	Low	Low	Low
Lian *et al*^16^	Low	High	Unclear	Unclear	Low	Low	Low
Meng *et al*^17^	High	High	Unclear	Unclear	High	High	Low
Morello *et al*^18^	High	Low	Low	Low	Unclear	Low	Low
Morello *et al*^19^	Low	High	Low	Low	Low	Low	Low
Morello *et al*^20^	High	High	Low	High	Low	Low	Low
Morello *et al*^21^	Unclear	High	Low	High	Low	Low	Low
Peng *et al*^22^	High	High	Unclear	Low	High	Low	Low
Suzuki *et al*^23^	High	High	Unclear	Unclear	Unclear	Low	Low
von Kodolitsch *et al*^24^	Unclear	Unclear	Unclear	High	Unclear	Low	Low
Wang *et al*^25^	High	High	Unclear	Low	High	Low	Low
Zhang *et al*^26^	Low	High	Unclear	High	Low	Low	Low

QUADAS-2Quality Assessment of Diagnostic Accuracy Studies 2

### Diagnostic performance of alternate biomarkers

The accuracy results (sensitivity, specificity, positive and negative predictive values, positive and negative likelihood ratios and area under the receiver operating characteristic (AUROC) of the 13 included studies are summarised in table 3 and online supplemental appendix S5. The AUROC for the biomarkers was generally modest, with high sensitivity with tight CIs achieved only when a threshold was used that resulted in low specificity. In general, the alternative biomarkers did not achieve the sensitivity and sensitivity of D-dimer reported in recent meta-analysis.^3^ There were two exceptions. Wang *et al*^25^ reported 99.1% sensitivity and 84.9% specificity for soluble ST2, but this differed markedly from the sensitivity of 58% and specificity of 70.8% reported by Morello *et al*.^21^ Chun and Siu^14^ reported 95.6% sensitivity and 56.1% specificity for neutrophil count, but this has not been confirmed by other studies. Accuracy improved in the event the biomarkers were combined with D-dimer but was not clearly superior to D-dimer alone.

**Table 3:** Summary of accuracy results (sensitivity, specificity and AUROC) along with their respective 95% CIs and cut-off values where provided for the biomarkers investigated in the 13 included cohort studies

Study	Biomarker	Sensitivity (%)	95% CI	Specificity (%)	95% CI	AUROC	95% CI	Cut-off*
Chun and Siu^14^	Neutrophil count (2–8 hours after symptom onset)	94.8	84.7 to 98.6	59.4	50 to 68.4	NR	NR	≥6.2×10^9^/L
	Neutrophil count (8–24 hours)	96.9	82 to 99.8	45	29.6 to 61.3	NR	NR	
	Neutrophil count (2–24 hours)	95.6	88.4 to 98.6	56.1	47.9 to 63.9	NR	NR	
	Neutrophil count (2–24 hours) and ADD-RS ≤1)	94.6	84.2 to 98.6	52.3	42.1 to 61.9	NR	NR	
Giachino *et al*^15^	MMP-8	100	93.2 to 100	9.5	3.9 to 18.5	0.75	NR	3.6 ng/mL
	MMP-9	96.2	86.8 to 99.5	16.2	8.7 to 26.6	0.7	NR	20 ng/mL
	D-dimer	97.6	87.4 to 99.9	32.8	21.3 to 46	0.87	0.8 to 0.94	500 ng/mL
	Log2 D-dimer and MMP-8	100	91.6 to 100	13.1	5.8 to 24.2	0.89	0.82 to 0.95	>0.77
Lian *et al*^16^	Acidic calponin	77.6	NR	87.7	NR	0.889	NR	6.96 ng/mL
	Acidic calponin+ascending aortic root dilation	83.7	NR	89.2	NR	0.927	NR	Calponin 6.96 ng/mL and diameter >40 mm
Meng *et al*^17^	Troponin	16.7	NR	76.7	NR	NR	NR	>0.04 µg/mL (old), ≥30 µg/L (new)
	D-dimer	100	NR	51.3	NR	NR	NR	>500 ng/mL
Morello *et al*^18^	LDH	44	37 to 51	73	69 to 76	0.61	0.57 to 0.66	450 U/L
Morello *et al*^19^	WBC count	67.3	57.7 to 75.9	59	55.5 to 62.5	0.69	0.63 to 0.74	>9×10^3^/μL
	Platelet count	68.2	58.6 to 76.7	56.2	52.7 to 59.7	0.64	0.58 to 0.69	>200×10^3^/μL
	Fibrinogen	50.9	41.2 to 60.2	63.6	60.2 to 67	0.62	0.55 to 0.68	<350 mg/dL
	≥1 alteration(s)	95.5	89.7 to 98.5	18.3	15.7 to 21.2	NR	NR	N/A
Morello *et al*^20^	Copeptin	78.8	70.1 to 85.6	74.6	68.3 to 80.1	0.81 (AAS) 0.83 (AAD)	0.75 to 0.86 (AAS) 0.77 to 0.88 (AAD)	14 pmol/L
	D-dimer	95.2	88.3 to 98.1	65.4	58.5 to 71.8	0.92	0.89 to 0.96	≥500 ng/mL
	Copeptin+D-dimer	95.2	88.3 to 98.1	46.6	39.7 to 53.7	0.92	0.88 to 0.95	D-dimer <500 ng/mL and copeptin <10 pmol/L
Morello *et al*^21^	sST2	58	47 to 68.4	70.8	64.1 to 76.9	0.675	0.61 to 0.736	39.8 ng/mL
						0.717 (when low pretest risk)	0.655 to 0.772	
	D-dimer	95.8	88.1 to 99.1	30.7	19.6 to 43.7	0.842	0.753 to 0.908	500 ng/mL
Peng *et al*^22^	α-SMA	54.29	NR	90.24	NR	0.62	0.49 to 0.76	49.62 ng/mL
	smMHC	68.57	NR	90.24	NR	0.81	0.71 to 0.91	2.11 ng/mL
	sELAF	82.86	NR	68.29	NR	0.82	0.73 to 0.91	97.07 ng/mL
	PC1	85.71	NR	75.61	NR	0.9	0.83 to 0.96	357.33 pg/mL
	1 variable positive	100	NR	53.66	NR	NR	NR	N/A
	2 variables positive	94.29	NR	85.37	NR	0.95	0.87 to 0.99	N/A
	3 variables positive	77.14	NR	95.12	NR	NR	NR	N/A
	All positive	51.43	NR	100	NR	NR	NR	N/A
	D-dimer	80	NR	90.21	NR	0.93	0.87 to 0.98	>2110 ng/mL
Suzuki *et al*^23^	Acidic calponin (initial 6 hours)	50	NR	87	NR	0.63	NR	2.8 ng/L
	Acidic calponin (initial 24 hours)	58	NR	72	NR	0.63	NR	2.3 ng/L
	Basic calponin (initial 6 hours)	63	NR	73	NR	0.67	NR	159 ng/L
	Basic calponin (initial 24 hours)	50	NR	66	NR	0.58	NR	139 ng/L
von Kodolitsch *et al*^24^	Leucocyte count	25.8	NR	77.9	NR	NR	NR	≥15×10^9^/L
Wang *et al*^25^	Soluble ST2	99.1	NR	84.9	NR	0.97	0.95 to 0.98	34.6 ng/mL
	Troponin	NR	NR	NR	NR	0.5	0.44 to 0.56	NR
	D-dimer	93.9	NR	78.5	NR	0.91	0.88 to 0.94	323 ng/mL
Zhang *et al*^26^	Neutrophil-to-lymphocyte ratio	76	71 to 81	79	74 to 83	0.845	0.816 to 0.871	NR
	D-dimer	74	69 to 79	76	72 to 80	0.822	0.792 to 0.850	NR

* Morello *et al*^18^ and Chun and Siu 2023^14^ used the standard laboratory cut-offs; Giachino *et al* 2013,^15^ Morello *et al* 2018,^20^ Morello *et al* 2020,^21^ Peng *et al* 2015,^22^ Wang *et al* 2018^25^ and Lian *et al* 2023^16^ all used accuracy data to determine the optimal cut-off and von Kodolitsch *et al* 2000^24^ and Zhang *et al* 2023^26^ did not record how the cut-off was reached.

ADD-RS, aortic dissection detection risk score; LDH, lactate dehydrogenase; MMP, matrix metalloproteinase; N/Anot availableNR, not reportedPC1, polycystin-1; sELAF, soluble elastin fragments; smMHC, smooth muscle myosin heavy chain; sST2, soluble suppression of tumourigenicity-2; WBC, white blood cellα-SMA, α-smooth muscle actin

## Discussion

### Summary of results

This systematic review has shown that biomarkers for AAS, other than D-dimer, currently have insufficient evidence of acceptable accuracy to support routine clinical use. We identified numerous studies evaluating many different biomarkers, but the quality and heterogeneity of the studies limited the conclusions we could draw. The estimates of sensitivity, specificity and AUROC from the cohort studies generally suggested poor diagnostic accuracy for AAS and thus these biomarkers have no current role in clinical practice.

We are aware of one additional review of alternative biomarkers for AAS.^64^ Chen *et al* concluded that microRNA biomarkers may have better specificity than D-dimer but current studies are insufficient. Their review included case-control studies, which are known to overestimate diagnostic accuracy,^10^ and undertook limited quality assessment of included studies, so any conclusions should be interpreted with caution.

### Interpretation of results

Soluble ST2 is primarily found in inflammatory processes and T-cell-mediated immune responses. The studies of soluble ST2 produced conflicting results, with Wang *et al*^25^ reporting diagnostic accuracy superior to that of D-dimer but Morello *et al*^21^ reporting modest accuracy. These differences could be attributed to differences in ethnicity and age across the two study populations. Further studies are required to determine accuracy in an appropriate cohort.

MMPs are key to aortic remodelling and part of the family of extracellular matrix markers. The study of Giachino *et al*^15^ suggested modest accuracy for AAS, but the combination of D-dimer and MMP-8 improved the sensitivity of D-dimer at the expense of specificity. Morello *et al*^20^ reported that copeptin, a biomarker released by the neurohypophysis in response to stress, provided suboptimal diagnostic accuracy for AAS. Suzuki *et al*^23^ reported that calponin, an analogue of cardiac troponin and released during muscle fibre apoptosis, had modest diagnostic accuracy for AAS. This was replicated by Lian *et al*,^16^ who demonstrated that including ascending aortic root dilation of >40 mm significantly increased the accuracy of calponin.

Peng *et al*^22^ focused on two distinct groups of biomarkers: smooth muscle biomarkers (α-SMA, smMHC and PC1) and extracellular matrix markers (sELAF). smMHC has been found to be released from damaged aortic medial muscle cells during aortic dissection, while PC1 plays a key role in stability and integrity of aortic vessel walls. sELAF is released on rupture of elastic vascular wall fibres. In isolation, none of these markers offered superior performance to D-dimer, but the combination of three of the biomarkers alongside D-dimer could offer better accuracy than D-dimer alone. This requires validation in a new cohort.

Some of the biomarkers evaluated for AAS are already used in routine clinical assessment for other conditions. Morello *et al*^19^ showed that WBC count, platelet count and fibrinogen are not accurate biomarkers for AAS but the modest diagnostic information they provide could be used in pretest probability assessment, while the study of von Kodolitsch *et al*^24^ suggested that leucocyte count provided no useful diagnostic information. Similarly, LDH was found to have poor diagnostic accuracy for AAS.^18^ The study of Zhang *et al*^26^ suggested that the neutrophil-to-lymphocyte ratio may have similar or superior accuracy to D-dimer in the diagnosis of AAS.^26^ This finding requires replication in other studies. Two studies of troponin showed no diagnostic value for AAS.^17 25^ Chun and Siu^14^ reported similar accuracy data to that of D-dimer, although these findings will require validation in further studies.

### Strengths and limitations of the systematic review

We used established and robust methods to ensure our review was comprehensive and involved objective assessment of study quality. However, it may have some limitations. Indexing and reporting of diagnostic studies may be suboptimal, especially if biomarker analysis is a secondary study objective, so we may have missed some potentially relevant data. The primary studies had important limitations, with insufficient numbers of cohort studies evaluating any individual biomarker to support meta-analysis and potential biases affecting patient selection and reference standard adjudication. There was also a lack of implementation studies, threshold rationale and economic data, thus making it difficult to draw conclusions regarding the practicality and affordability of clinical implementation of these alternative biomarkers. Furthermore, we noted that a number of our selected cohort studies reported high AAS prevalence, compared with an unselected population with possible AAS, such as reported in the Diagnosis of Acute Aortic Syndrome in the Emergency Department (DAShED) study.^65^ This likely represents selection of patients who received a definitive imaging reference standard, which limits the applicability of findings to the unselected general population.

The QUADAS-2 assessment of the reference standard did not differentiate between studies using AD and those using AAS as the reference standard. However, between 73% and 86% of cases of AAS were AD in the cohort studies that used AAS as the reference standard, so any differences between the studies using AD and AAS are likely to be modest and unlikely to impact our conclusions.

### Implications for policy, practice and future research

More research is needed on biomarkers for AAS. However, the low incidence of AAS presentations in the ED creates a substantial barrier to conducting adequately powered studies with robust designs. The researchers who undertook the studies included in this review deserve credit for their efforts, particularly the Italian researchers who provided 5 of the 13 studies. Our review highlights the need for other research teams to evaluate new biomarkers for AAS so we can determine whether findings are reproduced elsewhere. Case-control studies can provide initial data to identify biomarkers that show an association with AAS but cohort studies are required to provide data to guide clinical practice. Studies should ideally record clinical risk assessment, such as the ADD-RS, and measure D-dimer, to determine the additional contribution to diagnostic assessment of novel biomarkers. An optimal future study might involve recording the ADD-RS and taking blood for measurement of D-dimer and multiple alternative biomarkers from a large prospective cohort of patients with suspected AAS. Analysis could then determine whether and how alternative biomarkers provide additional diagnostic information beyond that provided by the ADD-RS and D-dimer.

## Conclusions

Currently available research is insufficient to recommend any biomarker as an alternative or in addition to D-dimer in the diagnostic assessment of AAS. Large cohort studies are required to evaluate multiple biomarkers in an appropriate population with suspected AAS.

## supplementary material

10.1136/emermed-2023-213772 online supplemental file 1

10.1136/emermed-2023-213772 online supplemental file 2

10.1136/emermed-2023-213772 online supplemental file 3

10.1136/emermed-2023-213772 online supplemental file 4

10.1136/emermed-2023-213772 online supplemental file 5

## Data Availability

All data relevant to the study are included in the article or uploaded as supplementary information.
