# Supplementary material for: Diagnostic accuracy of alternative biomarkers for acute aortic syndrome: a systematic review
Source: Emerg Med J. 2024 Aug 6;41(11):e213772. doi: 10.1136/emermed-2023-213772 (PMC11503200; doi:10.1136/emermed-2023-213772)
Supplement: online supplemental file 2 [file emermed-41-11-s002.pdf]

APPENDIX S2: LIST OF EXCLUDED STUDIES WITH RATIONALE

	**Authors, year**	**Reason for exclusion**
1.	Ahmadi et al., 2018[1]	Review (no primary data)
2.	Akutsu et al., 2005[2]	No alternative biomarkers
3.	Baez and Cochon, 2017[3]	Review (no primary data)
4.	Barry and Coughlan, 2019[4] (abstract)	No alternative biomarkers
5.	Cakir et al., 2021[5]	Case-control (control - healthy volunteers)
6.	Chen et al., 2023[6]	Review (no primary data)
7.	Cheng et al., 2020[7]	Case-control (control - not described)
8.	Deng et al., 2023[8]	No alternative biomarkers
9.	Derksen et al., 2018 (abstract)[9]	No alternative biomarkers
10.	Dong et al., 2017a[10]	Case-control (selected controls with alternative diagnoses or healthy volunteers)
11.	Dong et al., 2017b[11]	Case-control (selected controls with alternative diagnoses or healthy volunteers)
12.	Duceau et al., 2020[12]	No alternative biomarkers
13.	Eggebrecht et al., 2004[13]	Case-control (selected control with other pathology [chest-pain; pulmonary embolism acute myocardial infarction and asymptomatic with chronic aortic dissection] not suspected AAS)
14.	Ersel et al., 2010[14]	No alternative biomarkers
15.	Fan et al., 2010[15]	No alternative biomarkers
16.	Fletcher et al., 2021[16]	Case-control (selected controls with alternative diagnoses)
17.	Forrer et al., 2021[17]	Case-control (selected control with other pathology not suspected AAS)
18.	Gawinecka et al., 2021[18] (abstract)	Abstract of a full text study (Forrer et al., 2021[17])
19.	Giachino et al., 2014[19] (abstract)	No alternative biomarkers
20.	Goliopoulou et al., 2022[20] (abstract)	Case-control (controls - patients with dilated aorta or other cardiac surgery)
21.	Gorla et al., 2017a[21]	No alternative biomarkers
22.	Gorla et al.,2017b[22]	No alternative biomarkers
23.	Guo et al., 2013[23]	No alternative biomarkers

24.	Hagiwara et al., 2010[24] (abstract)	Case-control (controls -patients with other cardiovascular conditions)
25.	Hagiwara et al., 2013[25]	Case-control (selected control with other pathology not suspected AAS)
26.	Han et al., 2021[26]	Case-control (control - healthy volunteers)
27.	Hashemi et al., 2021[27] (abstract)	No alternative biomarkers
28.	Hazui et al., 2005[28]	Case-control (control - patients with acute myocardial infarction)
29.	Inaba et al., 2018[29] (abstract)	No alternative biomarkers
30.	Jiang et al., 2022[30]	Case-control (control - healthy volunteers)
31.	Kaito et al., 2022[31]	No alternative biomarkers
32.	Khan et al., 2023[32]	No alternative biomarkers
33.	Kodera and Kanda, 2016[33] (abstract)	No alternative biomarkers
34.	Konig et al., 2021[34]	Case-control (control - healthy volunteers)
35.	Konig et al., 2021 (abstract)[35]	Abstract of a full text study (Konig et al., 2021[34])
36.	Kotani et al., 2017[36]	No alternative biomarkers
37.	Lee et al., 2022[37]	No alternative biomarkers
38.	Levcik et al., 2013[38]	No alternative biomarkers
39.	Li et al., 2010[39] (abstract)	No alternative biomarkers
40.	Li et al., 2017[40]	No alternative biomarkers
41.	Li et al., 2018[41]	Case-control (control - healthy volunteers)
42.	Li et al., 2022[42]	Case-control (control - healthy volunteers)
43.	Liu et al., 2018[43]	Case-control (selected controls did not have symptoms suggesting AAS)
44.	Liu et al., 2022[44]	No alternative biomarkers
45.	Lovy et al., 2013[45]	No alternative biomarkers
46.	Lu et al., 2022[46]	Case-control (control - healthy volunteers)
47.	McLatchie et al., (unpublished)[47]	No alternative biomarkers
48.	Morello et al., 2017[48] (abstract)	No alternative biomarkers
49.	Morello et al., 2021[49]	No alternative biomarkers
50.	Morello et al., 2023[50]	All patients have AAS
51.	Nazerian et al., 2013[51] (abstract)	No alternative biomarkers
52.	Nazerian et al., 2014a[52]	No alternative biomarkers
53.	Nazerian et al., 2014b[53]	No alternative biomarkers

54.	Nazerian et al., 2018[54]	No alternative biomarkers
55.	Nazerian et al., 2018[55] (abstract)	No alternative biomarkers
56.	Ohle et al., 2017[56] (abstract)	Duplicate
57.	Ohle et al., 2018a[57] (abstract)	No alternative biomarkers
58.	Ohle et al., 2018b[58]	Case-control (unselected controls with suspected AAS)
59.	Ohle et al., 2019[59]	No alternative biomarkers
60.	Ohle et al., 2019[60] (abstract)	No alternative biomarkers
61.	Ohle et al., 2023[61]	No alternative biomarkers
62.	Ohlmann et al., 2006[62]	Case-control (unselected controls with suspected AAS)
63.	Okazaki et al., 2014[63]	Case-control (selected controls with other pathology not suspected AAS)
64.	Paige et al., 2020[64] (abstract)	Duplicate
65.	Pan et al., 2021[65]	Case-control (selected controls with alternative diagnoses or healthy volunteers)
66.	Qiming et al., 2010[66] (abstract)	No alternative biomarkers
67.	Reeps et al., 2010[67]	All patients have AAS
68.	Rotella et al., 2018[68]	No alternative biomarkers
69.	Sakamoto et al., 2011[69]	No alternative biomarkers
70.	Sakamoto et al., 2016[70]	Population/cases are stroke not suspected AAS
71.	Sbarouni et al., 2007[71]	Case-control (selected controls with alternative diagnoses or healthy volunteers)
72.	Sbarouni et al., 2015[72]	Case-control (selected controls with alternative diagnoses or healthy volunteers)
73.	Sbarouni et al., 2018[73]	Case-control (selected controls with alternative diagnoses or healthy volunteers)
74.	Shao et al., 2014[74]	No alternative biomarkers
75.	Shinohara et al., 2003[75]	Case-control (control - healthy volunteers and patients with acute myocardial infarction)
76.	Shirakabe et al., 2008[76]	No alternative biomarkers
77.	Song et al., 2022[77]	Case-control (control - healthy volunteers and patients with acute coronary syndrome)
78.	Song et al., 2023[78]	No alternative biomarkers
79.	Spinner et al., 2006[79] (abstract)	No alternative biomarkers

80.	Stanojlovic et al., 2013[80] (abstract)	No alternative biomarkers
81.	Suzuki et al., 1996[81]	Case control (controls with myocardial infarction)
82.	Suzuki et al., 2009[82]	No alternative biomarkers
83.	Suzuki et al., 2011[83]	Case-control (selected controls with alternative diagnoses)
84.	Tokuda et al., 2016[84]	No alternative biomarkers
85.	Tokuda et al., 2018[85]	No alternative biomarkers
86.	Wagner et al., 2002[86]	Case-control (selected controls with alternative diagnoses)
87.	Wang et al., 2018[87]	No alternative biomarkers
88.	Weber et al., 2003[88]	No alternative biomarkers
89.	Wei et al., 2012[89] (abstract)	All patients have AAS
90.	Wiegand et al., 2007[90]	All patients have AAS
91.	Wilson et al., 2016[91] (abstract)	No alternative biomarkers
92.	Xiao et al., 2016[92]	Case-control (unselected controls with suspected AAS)
93.	Xu et al., 2017[93]	Case-control (control - not described but appear to be healthy volunteers)
94.	Xu et al., 2022[94]	Case-control (controls with chest pain without acute aortic dissection)
95.	Xue et al., 2007[95]	No alternative biomarkers
96.	Yamashita et al., 2018[96]	No alternative biomarkers
97.	Yoshimuta et al., 2015[97]	Population/cases are stroke not suspected AAS
98.	Yuan et al., 2011[98]	Case-control (selected controls with alternative diagnoses)
99.	Zeng et al., 2020[99]	Case-control (control - healthy volunteers)
100.	Zhang et al., 2023[100]	Case-control (unselected controls with suspected AAS)
101.	Zhang et al., 2023[101]	Duplicate
102.	Zhao et al., 2020[102]	Case-control (controls with myocardial infarction and pulmonary embolism)
103.	Zheng et al., 2012[103] (abstract)	Case-control (control - healthy volunteers and patients with alternate diagnoses) (abstract only)
104.	Zitek et al., 2022[104]	No alternative biomarkers

REFERENCES

1. Ahmadi I, Qavam SM, Sayehmiri K, et al. Investigating the efficiency of D-Dimer test in diagnosis of aortic dissection: A systematic study and meta-analysis. *International Cardiovascular Research Journal* 2018;12(4):142-47.
2. Akutsu K, Sato N, Yamamoto T, et al. A rapid bedside D-dimer assay (cardiac D-dimer) for screening of clinically suspected acute aortic dissection. *Circulation Journal : Official Journal of the Japanese Circulation Society* 2005;69(4):397-403.
3. Baez AA, Cochon L. Improved rule-out diagnostic gain with a combined aortic dissection detection risk score and D-dimer Bayesian decision support scheme. *Journal of Critical Care* 2017;37:56-59. doi: https://dx.doi.org/10.1016/j.jcrc.2016.08.007
4. Barry I, Coughlan F. Computed tomography thoracic angiography referred from a tertiary ED: An audit to determine if this test is being utilised optimally in the investigation of acute aortic dissection. *Journal of Medical Imaging and Radiation Oncology* 2019;63(Supplement 1):154-55.
5. Cakir A, Payza U, Aksun S, et al. Validity of signal peptide-cub-egf domain-containing protein-1 (Scube-1) in the diagnosis of aortic dissection. *Signa Vitae* 2021;17(1):112-16.
6. Chen H, Li Y, Li Z, et al. Diagnostic biomarkers and aortic dissection: a systematic review and meta-analysis. *BMC Cardiovascular Disorders* 2023;23(1):497. doi: https://dx.doi.org/10.1186/s12872-023-03448-9
7. Cheng N, Wang H, Zhang W, et al. Comparative Proteomic Investigation of Plasma Reveals Novel Potential Biomarker Groups for Acute Aortic Dissection. *Disease Markers* 2020;2020:4785068. doi: https://dx.doi.org/10.1155/2020/4785068
8. Deng L, Xia Q, Diao L, et al. Aortic Dissection Detection Risk Score and D-Dimer for Acute Aortic Syndromes in the Chinese Population: Exploration of Optimal Thresholds and Integrated Diagnostic Value. *Journal of Cardiovascular Translational Research* 2023;16(4):886-95. doi: https://dx.doi.org/10.1007/s12265-023-10354-0
9. Derksen B, Glober N, Darocki M, et al. Is the highly sensitive HemosIL D-dimer a valuable screening tool to rule out aortic dissection? *Academic Emergency Medicine* 2018;25(Supplement 1):S197-S98.
10. Dong J, Bao J, Feng R, et al. Circulating microRNAs: a novel potential biomarker for diagnosing acute aortic dissection. *Scientific Reports* 2017;7(1):12784. doi: https://dx.doi.org/10.1038/s41598-017-13104-w
11. Dong J, Duan X, Feng R, et al. Diagnostic implication of fibrin degradation products and D-dimer in aortic dissection. *Scientific Reports* 2017;7:43957. doi: https://dx.doi.org/10.1038/srep43957

12. Duceau B, Alsac JM, Bellenfant F, et al. Prehospital triage of acute aortic syndrome using a machine learning algorithm. *The British Journal of Surgery* 2020;107(8):995-1003. doi: https://dx.doi.org/10.1002/bjs.11442
13. Eggebrecht H, Naber CK, Bruch C, et al. Value of plasma fibrin D-dimers for detection of acute aortic dissection. *Journal of the American College of Cardiology* 2004;44(4):804-9.
14. Ersel M, Aksay E, Kiyan S, et al. Can D-dimer testing help emergency department physicians to detect acute aortic dissections? *Anadolu kardiyoloji dergisi : AKD = the Anatolian Journal of Cardiology* 2010;10(5):434-9. doi: https://dx.doi.org/10.5152/akd.2010.142
15. Fan Q-k, Wang W-w, Zhang Z-l, et al. Evaluation of D-dimer in the diagnosis of suspected aortic dissection. *Clinical Chemistry and Laboratory Medicine* 2010;48(12):1733-7. doi: https://dx.doi.org/10.1515/CCLM.2010.337
16. Fletcher A, Syed M, Iskander Z, et al. Plasma desmosine as a biomarker in acute aortic syndrome. *European Heart Journal* 2021;42(Supplement_1):ehab724. 2011.
17. Forrer A, Schoenrath F, Torzewski M, et al. Novel Blood Biomarkers for a Diagnostic Workup of Acute Aortic Dissection. *Diagnostics (Basel, Switzerland)* 2021;11(4) doi: https://dx.doi.org/10.3390/diagnostics11040615
18. Gawinecka J, Forrer A, Schonrath F, et al. Novel biomarkers in diagnostic workup of acute aortic dissection. *Atherosclerosis* 2021;331:e248.
19. Giachino F, Bono A, Castelli M, et al. Diagnostic performance of D-dimer in patients with history of aortic dissection. *Giornale Italiano di Cardiologia* 2014;2):e161.
20. Goliopoulou A, Oikonomou E, Antonopoulos A, et al. Expression of Tissue microRNAs in Ascending Aortic Aneurysms and Dissections. *Angiology* 2022:33197221098295.
21. Gorla R, Erbel R, Kahlert P, et al. Accuracy of a diagnostic strategy combining aortic dissection detection risk score and D-dimer levels in patients with suspected acute aortic syndrome. *European Heart Journal Acute Cardiovascular Care* 2017;6(5):371-78. doi: https://dx.doi.org/10.1177/2048872615594497
22. Gorla R, Erbel R, Kahlert P, et al. Diagnostic role and prognostic implications of D-dimer in different classes of acute aortic syndromes. *European Heart Journal Acute Cardiovascular Care* 2017;6(5):379-88. doi: https://dx.doi.org/10.1177/2048872615594500
23. Guo Z-g, Ma Q-b, Zheng Y-a, et al. [The value of D-dimer for etiological diagnosis of mortal chest pain: an analysis of 438 cases]. *Zhonghua wei zhong bing ji jiu yi xue* 2013;25(11):655-9. doi: https://dx.doi.org/10.3760/cma.j.issn.2095-4352.2013.11.005
24. Hagiwara A, Sakamoto D, Sasaki R, et al. Diagnosis of acute aortic dissection using a fibrinolytic marker. *Critical Care Medicine* 2010;12):A178.
25. Hagiwara A, Shimbo T, Kimira A, et al. Using fibrin degradation products level to facilitate diagnostic evaluation of potential acute aortic dissection. *Journal of Thrombosis and Thrombolysis* 2013;35(1):15-22. doi: https://dx.doi.org/10.1007/s11239-012-0779-6

26. Han C, Liu Q, Li Y, et al. S100A1 as a potential biomarker for the diagnosis of patients with acute aortic dissection. *The Journal of International Medical Research* 2021;49(4):3000605211004512. doi: https://dx.doi.org/10.1177/03000605211004512
27. Hashemi M, Zagroba S, Zitek T. 299 The Diagnostic Value of D-dimer Levels for the Exclusion of Aortic Dissection. *Annals of Emergency Medicine* 2021;78(4 Supplement):S121-S22.
28. Hazui H, Fukumoto H, Negoro N, et al. Simple and useful tests for discriminating between acute aortic dissection of the ascending aorta and acute myocardial infarction in the emergency setting. *Circulation Journal : Official Journal of the Japanese Circulation Society* 2005;69(6):677-82.
29. Inaba H, Yamashita A, Yoshita Y, et al. Do emergency medical service systems properly asssess the risk of acute aortic syndome and transport the patients to the right hospitals? Impact of ems assessment and transportation in outcomes. *Cardiology (Switzerland)* 2018;140(Supplement 1):299.
30. Jiang Y, Tang X, Wang Y, et al. Serum Oxylipin Profiles Identify Potential Biomarkers in Patients with Acute Aortic Dissection. *Metabolites* 2022;12(7) (no pagination)
31. Kaito D, Yamamoto R, Nakama R, et al. D-dimer for screening of aortic dissection in patients with ST-elevation myocardial infarction. *American Journal of Emergency Medicine* 2022;59:146-51.
32. Khan A, Masoura C, Mitra A, et al. Accuracy of aortic dissection detection risk score and D-dimer to rule out acute aortic syndromes in the emergency department. *Critical Care* 2023;27(Supplement 1) doi: https://dx.doi.org/10.1186/s13054-023-04377-x
33. Kodera S, Kanda J. Comparison aortic dissection detection risk score and d-dimer in diagnosis of acute aortic dissection. *Cardiology (Switzerland)* 2016;134(Supplement 1):349.
34. Konig KC, Lahm H, Dresen M, et al. Aggrecan: a new biomarker for acute type A aortic dissection. *Scientific Reports* 2021;11(1):10371. doi: https://dx.doi.org/10.1038/s41598-021-89653-y
35. Konig C, Lahm H, DreBen M, et al. Aggrecan: A new biomarker for acute thoracic aortic dissection. *Thoracic and Cardiovascular Surgeon Conference: 50th Annual Meeting of the German Society for Thoracic and Cardiovascular Surgery, DGTHG* 2021;69(SUPPL 1)
36. Kotani Y, Toyofuku M, Tamura T, et al. Validation of the diagnostic utility of D-dimer measurement in patients with acute aortic syndrome. *European Heart Journal Acute Cardiovascular Care* 2017;6(3):223-31. doi: https://dx.doi.org/10.1177/2048872616652261
37. Lee D, Kim YW, Kim TY, et al. Age-Adjusted D-Dimer in Ruling Out Acute Aortic Syndrome. *Emergency Medicine International* 2022;2022:6864756. doi: https://dx.doi.org/10.1155/2022/6864756

38. Levcik M, Kettner J, Jabor A, et al. Utility of plasma D-dimer levels in the diagnosis of acute aortic dissection. *Cor et Vasa* 2013;55(6):e510-e14. doi: https://dx.doi.org/10.1016/j.crvasa.2013.04.009
39. Li W, Fan X, Xu J, et al. The value of D-dimer in acute aortic dissection: The experience of China. *Atherosclerosis Supplements* 2010;11(2):197.
40. Li W, Huang B, Tian L, et al. Admission D-dimer testing for differentiating acute aortic dissection from other causes of acute chest pain. *Archives of Medical Science : AMS* 2017;13(3):591-96. doi: https://dx.doi.org/10.5114/aoms.2017.67280
41. Li T, Jiang B, Li X, et al. Serum matrix metalloproteinase-9 is a valuable biomarker for identification of abdominal and thoracic aortic aneurysm: a case-control study. *BMC Cardiovascular Disorders* 2018;18(1):202. doi: https://dx.doi.org/10.1186/s12872-018-0931-0
42. Li T, Zhou Y, Li D, et al. The role of genome-scale leukocyte long noncoding RNA in identifying acute aortic dissection. *Signa Vitae* 2022;18(3):101-10.
43. Liu X, Zheng X, Su X, et al. Plasma Resistin Levels in Patients with Acute Aortic Dissection: A Propensity Score-Matched Observational Case-Control Study. *Medical Science Monitor : International Medical Journal of Experimental and Clinical Research* 2018;24:6431-37. doi: https://dx.doi.org/10.12659/MSM.909469
44. Liu W-T, Lin C-S, Tsao T-P, et al. A Deep-Learning Algorithm-Enhanced System Integrating Electrocardiograms and Chest X-rays for Diagnosing Aortic Dissection. *The Canadian Journal of Cardiology* 2022;38(2):160-68. doi: https://dx.doi.org/10.1016/j.cjca.2021.09.028
45. Lovy AJ, Bellin E, Levsky JM, et al. Preliminary development of a clinical decision rule for acute aortic syndromes. *The American Journal of Emergency Medicine* 2013;31(11):1546-50. doi: https://dx.doi.org/10.1016/j.ajem.2013.06.005
46. Lu P, Feng X, Li R, et al. A Novel Serum Biomarker Model to Discriminate Aortic Dissection from Coronary Artery Disease. *Disease Markers* 2022;2022:9716424.
47. McLatchie R, Reed MJ, Freeman N, et al. Diagnosis of Acute Aortic Syndrome in the Emergency Department (DAShED) study: an observational cohort study of people attending the emergency department with symptoms consistent with acute aortic syndrome. *Emergency Medicine Journal* 2023:Published Online First: 09 November 2023. doi: 10.1136/emermed-2023-213266
48. Morello F, Nazerian P, Mueller C, et al. Rule-out of acute aortic syndrome by integration of the aortic dissection detection risk score plus d-dimer: Preliminary data from the ADvISED prospective multicenter study. *European Heart Journal* 2017;38(Supplement 1):1264.
49. Morello F, Bima P, Pivetta E, et al. Development and Validation of a Simplified Probability Assessment Score Integrated With Age-Adjusted d-Dimer for Diagnosis of Acute Aortic

Syndromes. *Journal of the American Heart Association* 2021;10(3):e018425. doi: https://dx.doi.org/10.1161/JAHA.120.018425

50. Morello F, Santoro M, Giachino F, et al. Pre-Test Probability Assessment and d-Dimer Based Evaluation in Patients with Previous Acute Aortic Syndrome. *Medicina (Kaunas, Lithuania)* 2023;59(3) doi: https://dx.doi.org/10.3390/medicina59030548
51. Nazerian P, Morello F, Vanni S, et al. Combined use of a standardized risk score and d-dimer to rule out acute aortic dissection in the emergency department. *European Heart Journal* 2013;1):1113-14.
52. Nazerian P, Giachino F, Vanni S, et al. Diagnostic performance of the aortic dissection detection risk score in patients with suspected acute aortic dissection. *European Heart Journal Acute Cardiovascular Care* 2014;3(4):373-81. doi: https://dx.doi.org/10.1177/2048872614527010
53. Nazerian P, Morello F, Vanni S, et al. Combined use of aortic dissection detection risk score and D-dimer in the diagnostic workup of suspected acute aortic dissection. *International Journal of Cardiology* 2014;175(1):78-82. doi: https://dx.doi.org/10.1016/j.ijcard.2014.04.257
54. Nazerian P, Mueller C, Soeiro AdM, et al. Diagnostic Accuracy of the Aortic Dissection Detection Risk Score Plus D-Dimer for Acute Aortic Syndromes: The ADvISED Prospective Multicenter Study. *Circulation* 2018;137(3):250-58. doi: https://dx.doi.org/10.1161/CIRCULATIONAHA.117.029457
55. Nazerian P, Vanni S, Mueller C, et al. Diagnostic performance of enlarged mediastinum on chest X ray in patients with suspected acute aortic dissection. the ADVISED-CXR multicenter prospective study. *European Heart Journal: Acute Cardiovascular Care* 2018;7(1 Supplement 1):338.
56. Ohle R, Um SW, Wells GA, et al. High-risk clinical features for acute aortic syndrome. *Academic Emergency Medicine* 2017;24(Supplement 1):S88.
57. Ohle R, McIsaac S, Perry JJ. A RAPID bedside approach to ruling out acute aortic dissection. *Canadian Journal of Emergency Medicine* 2018;20(Supplement 1):S17-S18.
58. Ohle R, Um J, Anjum O, et al. High Risk Clinical Features for Acute Aortic Dissection: A Case-Control Study. *Academic Emergency Medicine* 2018;25(4):378-87.
59. Ohle R, Anjum O, Bleeker H, et al. What Is the Specificity of the Aortic Dissection Detection Risk Score in a Low-prevalence Population? *Academic Emergency Medicine : Official Journal of the Society for Academic Emergency Medicine* 2019;26(6):632-38. doi: https://dx.doi.org/10.1111/acem.13634
60. Ohle R, Fortino N, Montpellier O, et al. Prospective pilot implementation of a clinical decision aid for acute aortic syndrome. *Canadian Journal of Emergency Medicine* 2019;21(Supplement 1):S98.

61. Ohle R, McIsaac S, Van Drusen M, et al. Evaluation of the Canadian Clinical Practice Guidelines Risk Prediction Tool for Acute Aortic Syndrome: The RIPP Score. *Emergency Medicine International* 2023;2023:6636800. doi: https://dx.doi.org/10.1155/2023/6636800
62. Ohlmann P, Faure A, Morel O, et al. Diagnostic and prognostic value of circulating D-Dimers in patients with acute aortic dissection. *Critical Care Medicine* 2006;34(5):1358-64.
63. Okazaki T, Yamamoto Y, Yoda K, et al. The ratio of D-dimer to brain natriuretic peptide may help to differentiate between cerebral infarction with and without acute aortic dissection. *Journal of the Neurological Sciences* 2014;340(1-2):133-8. doi: https://dx.doi.org/10.1016/j.jns.2014.03.011
64. Paige B, Maeng A, Savage D, et al. Validation of the Canadian clinical practice guideline clinical decision aid for acute aortic syndrome. *Canadian Journal of Emergency Medicine* 2020;22(Supplement 1):S43-S44.
65. Pan X, Zhou Y, Yang G, et al. Lysophosphatidic Acid May Be a Novel Biomarker for Early Acute Aortic Dissection. *Frontiers in Surgery* 2021;8:789992. doi: https://dx.doi.org/10.3389/fsurg.2021.789992
66. Qiming L, Ming T, Shenghua Z, et al. Analysis on early diagnosis grading model of acute aortic dissection. *Heart* 2010;3):A187.
67. Reeps C, Pelisek J, Bundschuh RA, et al. Imaging of acute and chronic aortic dissection by 18F-FDG PET/CT. *Journal of Nuclear Medicine* 2010;51(5):686-91.
68. Rotella J-A, Goel V, Chan T, et al. Aortic dissection detection risk score has high sensitivity with moderate inter-rater reliability. *Emergency Medicine Australasia : EMA* 2018;30(5):720-21. doi: https://dx.doi.org/10.1111/1742-6723.13152
69. Sakamoto K, Yamamoto Y, Okamatsu H, et al. D-dimer is helpful for differentiating acute aortic dissection and acute pulmonary embolism from acute myocardial infarction. *Hellenic Journal of Cardiology : HJC = Hellenike kardiologike epitheorese* 2011;52(2):123-7.
70. Sakamoto Y, Koga M, Ohara T, et al. Frequency and Detection of Stanford Type A Aortic Dissection in Hyperacute Stroke Management. *Cerebrovascular Diseases (Basel, Switzerland)* 2016;42(1-2):110-6. doi: https://dx.doi.org/10.1159/000445528
71. Sbarouni E, Georgiadou P, Marathias A, et al. D-dimer and BNP levels in acute aortic dissection. *International Journal of Cardiology* 2007;122(2):170-2.
72. Sbarouni E, Georgiadou P, Analitis A, et al. High neutrophil to lymphocyte ratio in type A acute aortic dissection facilitates diagnosis and predicts worse outcome. *Expert Review of Molecular Diagnostics* 2015;15(7):965-70. doi: https://dx.doi.org/10.1586/14737159.2015.1042367
73. Sbarouni E, Georgiadou P, Kosmas E, et al. Platelet to lymphocyte ratio in acute aortic dissection. *Journal of Clinical Laboratory Analysis* 2018;32(7):e22447. doi: https://dx.doi.org/10.1002/jcla.22447

74. Shao N, Xia S, Wang J, et al. The role of D-dimers in the diagnosis of acute aortic dissection. *Molecular Biology Reports* 2014;41(10):6397-403. doi: https://dx.doi.org/10.1007/s11033-014-3520-z
75. Shinohara T, Suzuki K, Okada M, et al. Soluble elastin fragments in serum are elevated in acute aortic dissection. *Arteriosclerosis, Thrombosis, and Vascular Biology* 2003;23(10):1839-44.
76. Shirakabe A, Hata N, Yokoyama S, et al. Diagnostic score to differentiate acute aortic dissection in the emergency room. *Circulation Journal : Official Journal of the Japanese Circulation Society* 2008;72(6):986-90.
77. Song R, Xu N, Luo L, et al. Diagnostic Value of Aortic Dissection Risk Score, Coagulation Function, and Laboratory Indexes in Acute Aortic Dissection. *BioMed Research International* 2022;2022:7447230.
78. Song DH, Choi JH, Lee JY. Predicting acute aortic syndrome using aortic dissection detection risk score, D-dimer, and X-ray. *Heliyon* 2023;9(10):e20578. doi: https://dx.doi.org/10.1016/j.heliyon.2023.e20578
79. Spinner T, Spes C, Mudra H. Elevated d-dimer at acute chest pain: Pulmonary embolism or aortic dissection?. [German]. *Intensivmedizin und Notfallmedizin* 2006;43(7):570-74.
80. Stanojlovic T, Pavlovic MP, Ciric-Zdravkovic SCZ, et al. P468 Utility of D-dimer testing in ruling out the diagnosis of acute aortic syndrome. *European Heart Journal Acute Cardiovascular Care* 2013;2(1)
81. Suzuki T, Katoh H, Watanabe M, et al. Novel biochemical diagnostic method for aortic dissection: results of a prospective study using an immunoassay of smooth muscle myosin heavy chain. *Circulation* 1996;93(6):1244-49.
82. Suzuki T, Distante A, Zizza A, et al. Diagnosis of acute aortic dissection by D-dimer: the International Registry of Acute Aortic Dissection Substudy on Biomarkers (IRAD-Bio) experience. *Circulation* 2009;119(20):2702-7. doi: https://dx.doi.org/10.1161/CIRCULATIONAHA.108.833004
83. Suzuki T, Trimarchi S, Sawaki D, et al. Circulating transforming growth factor-beta levels in acute aortic dissection. *Journal of the American College of Cardiology* 2011;58(7):775-75.
84. Tokuda N, Koga M, Ohara T, et al. Urgent detection of acute type a aortic dissection in ischemic stroke or TIA. *Stroke Conference: American Heart Association/American Stroke Association* 2016;47(SUPPL. 1)
85. Tokuda N, Koga M, Ohara T, et al. Urgent Detection of Acute Type A Aortic Dissection in Hyperacute Ischemic Stroke or Transient Ischemic Attack. *Journal of Stroke and Cerebrovascular Diseases : the Official Journal of National Stroke Association* 2018;27(8):2112-17. doi: https://dx.doi.org/10.1016/j.jstrokecerebrovasdis.2018.03.010
86. Wagner A, Domanovits H, Holzer M, et al. Plasma endothelin in patients with acute aortic disease. *Resuscitation* 2002;53(1):71-76.

87. Wang D, Wang Z-Y, Wang J-F, et al. Values of aortic dissection detection risk score combined with ascending aorta diameter >40 mm for the early identification of type A acute aortic dissection. *Journal of Thoracic Disease* 2018;10(3):1815-24. doi: https://dx.doi.org/10.21037/jtd.2018.02.42
88. Weber T, Hogler S, Auer J, et al. D-dimer in acute aortic dissection. *Chest* 2003;123(5):1375-8.
89. Wei L, Faquan L, Jiumei C. Diagnostic mode for early diagnosis of acute aortic dissection. *Heart* 2012;2):E132.
90. Wiegand J, Koller M, Bingisser R. Does a negative D-dimer test rule out aortic dissection? *Swiss Medical Weekly* 2007;137(31-32):462.
91. Wilson S, Kinni H, Smoot T, et al. Overutilization of computed tomography angiography for acute aortic dissection: Identifying additional need for a reliable screening biomarker. *Academic Emergency Medicine* 2016;1):S56-S57.
92. Xiao Z, Xue Y, Yao C, et al. Acute Aortic Dissection Biomarkers Identified Using Isobaric Tags for Relative and Absolute Quantitation. *BioMed Research International* 2016;2016:6421451. doi: https://dx.doi.org/10.1155/2016/6421451
93. Xu Z, Wang Q, Pan J, et al. Characterization of serum miRNAs as molecular biomarkers for acute Stanford type A aortic dissection diagnosis. *Scientific Reports* 2017;7(1):13659. doi: https://dx.doi.org/10.1038/s41598-017-13696-3
94. Xu Z, Wei M, Guo X, et al. Changes of Serum D-Dimer, NT-proBNP, and Troponin I Levels in Patients with Acute Aortic Dissection and the Clinical Significance. *Evidence-Based Complementary & Alternative Medicine: eCAM* 2022;2022:8309505.
95. Xue C, Li Y. Value of D-Dimers in patients with acute aortic dissection. *Journal of Nanjing Medical University* 2007;21(2):86-88.
96. Yamashita A, Maeda T, Kita Y, et al. The impact of prehospital assessment and EMS transport of acute aortic syndrome patients. *The American Journal of Emergency Medicine* 2018;36(7):1188-94. doi: https://dx.doi.org/10.1016/j.ajem.2017.12.005
97. Yoshimuta T, Yokoyama H, Okajima T, et al. Impact of Elevated D-Dimer on Diagnosis of Acute Aortic Dissection With Isolated Neurological Symptoms in Ischemic Stroke. *Circulation Journal : Official Journal of the Japanese Circulation Society* 2015;79(8):1841-5. doi: https://dx.doi.org/10.1253/circj.CJ-15-0050
98. Yuan S-M, Shi Y-H, Wang J-J, et al. Elevated plasma D-dimer and hypersensitive C-reactive protein levels may indicate aortic disorders. *Revista brasileira de cirurgia cardiovascular : orgao oficial da Sociedade Brasileira de Cirurgia Cardiovascular* 2011;26(4):573-81.
99. Zeng Q, Rong Y, Li D, et al. Identification of Serum Biomarker in Acute Aortic Dissection by Global and Targeted Metabolomics. *Annals of Vascular Surgery* 2020;68:497-504. doi: https://dx.doi.org/10.1016/j.avsg.2020.06.026

100. Zhang D, Zhao X, Wang B, et al. Circulating exosomal miRNAs as novel biomarkers for acute aortic dissection: A diagnostic accuracy study. *Medicine* 2023;102(30):e34474. doi: https://dx.doi.org/10.1097/MD.0000000000034474
101. Zhang H, Yuan N, Guo J, et al. Comparisons of potential values of D-dimer and the neutrophil-to-lymphocyte ratio in patients with suspected acute aortic syndrome. *American Journal of Emergency Medicine* 2023;69:44-51. doi: https://dx.doi.org/10.1016/j.ajem.2023.03.059
102. Zhao G, Zhao Y, Zhang H. Value of duration of chest pain, troponin, and D-dimer in differentiating acute high-risk chest pain patient. *Acta Medica Mediterranea* 2020;36(3):1587-91.
103. Zheng Z, Zi Y, Jialin Y, et al. Value of D-dimer for detection of acute aortic dissection. *Heart* 2012;2):E268.
104. Zitek T, Hashemi M, Zagroba S, et al. A Retrospective Analysis of Serum D-Dimer Levels for the Exclusion of Acute Aortic Dissection. *Open Access Emergency Medicine* 2022;14:367-73.
