# Supplementary material for: Diagnostic accuracy of alternative biomarkers for acute aortic syndrome: a systematic review
Source: Emerg Med J. 2024 Aug 6;41(11):e213772. doi: 10.1136/emermed-2023-213772 (PMC11503200; doi:10.1136/emermed-2023-213772)
Supplement: online supplemental file 3 [file emermed-41-11-s003.pdf]

APPENDIX S3: Summary of the 38 excluded case-control studies

Lead Author	Year of Publication	Country	Cases	Controls	Case Selection	Control Selection	Biomarkers Evaluated	Reference Standard
Cheng N(1)	2020	China	20 AAD	20 Unspecified Controls	Patients diagnosed with AAD within set temporal boundaries	Unclear	Proteomic Investigation for Novel Biomarkers	Confirmed on Imaging
Dong J(2)	2017	China	63 (37 AD and 26 subacute AD)	40 (23 CP with other diagnosis, 17 healthy controls)	Patients diagnosed with AAD within set temporal boundaries	Patients diagnosed with alternative cause of CP within set temporal boundaries. Unclear how healthy controls selected	Micro-Ribonucleic Acids (MicroRNAs) and D-Dimer	Confirmed on Imaging
Dong J(3)	2017	China	202 AD patients	150 with other cardiovascular disease, 27 healthy people	Patients diagnosed with AAD within set temporal boundaries	Unclear	D-Dimer and Fibrin Degradation Products (FDP)	Confirmed on Imaging
Eggebrecht H(4)	2004	Germany and The Netherlands	16 AAD	16 PE, 16 MI, 16 non-cardiac pain, 32 chronic stable AD	Consecutive CP patients	Consecutive CP patients with alternative diagnosis, unclear how patients with chronic stable AD were selected	D-dimer, White Blood Cell Count (WBC), C-Reactive Protein (CRP) and fibrinogen	Confirmed on Imaging

Forrer A(5)	2021	Switzerland	29 AAD	121 (30 Non-Cardiac CP, 30 thoracic aortic aneurysm, 31 MI, 30 PE)	Consecutive patients diagnosed with AAD	Consecutive patients diagnosed with thoracic aortic aneurysm. MI patients selected from SPUM-ACS cohort. PE patients selected from SWITCO-65+ cohort.	Troponin, D-Dimer, Interleukin-10 (IL-10), Interleukin-6 (IL-6), Plasminogen Activator Inhibitor-1 (PAI1), Interleukin-1 receptor antagonist (IL-1ra), Insulin-Like Growth Factor Binding Protein-1 (IGFBP1)	Confirmed on Imaging
Hagiwara A(6)	2013	Japan	96	776 (187 MI, 142 Angina Pectoris, 353 CVA, 94 TIA)	Consecutive patients diagnosed with AAD	Consecutive patients during same period diagnosed with MI, Angina Pectoris, CVA and TIA	Fibrin Degradation Products and D-Dimer	Confirmed on Imaging
Han C(7)	2021	China	78	77 (Healthy)	Patients diagnosed with AAD within set temporal boundaries	Healthy controls selected within the same temporal boundaries from outpatient physical examinations	S100A1, D-Dimer, High-Sensitivity C-Reactive Protein (hs-CRP), Troponin	Confirmed on Imaging
Konig K(8)	2021	Germany	33	43 (13 MI, 18 thoracic aneurysm, 12 healthy volunteers)	Patients with AAD admitted for surgery non-consecutively selected within set temporal boundaries	Unclear	Aggrecan, Creatine Kinase Myocardial Band (CKMB), Troponin	Unclear
Li T(9)	2018	China	88	88 (Healthy)	Patients diagnosed with AAD within set	Unclear	Plasma Matrix Metalloproteinase 9 (MMP-9)	Confirmed on Imaging

					temporal boundaries		and Toll-Like Receptor 4 (TLR-4)	
Liu X(10)	2018	China	43	43 healthy controls	Unclear	Case-matched healthy individuals with normal physical and echocardiographic findings	Plasma Resistin	Confirmed on Imaging
Ohle R(11)	2018	Canada	194	776 nonspecific CP	Patients diagnosed with AAD within set temporal boundaries	Patients suspected of AAD but who received an alternative diagnosis in ED within set temporal boundaries	Creatinine, White Blood Cell Count and Liver Function Tests (LFTs)	Confirmed on Imaging
Ohlmann P(12)	2006	France	94	94 with AAD ruled out	Consective patients diagnosed with AAD	Case-matched patients with clinical suspicion for AAD but diagnosed otherwise	CRP, Troponin, Lactate Dehydrogenase (LDH), Leukocyte Count, D-Dimer	Confirmed on Imaging
Okazaki T(13)	2014	Japan	15	115 Acute CVA	Consecutive patients diagnosed with AAD	Consecutive patients diagnosed with CVA	D-Dimer, Brain Natriuretic Peptide (BNP) and D-Dimer:BNP Ratio	Confirmed on Imaging
Pan X(14)	2021	China	86 AAD	118 (60 MI, 28 PE, 30 Healthy Volunteers)	Consecutive patients presenting with CP and diagnosed with AAD	Consecutive CP patients with alternative diagnosis, unclear how healthy	Lysophosphatidic Acid (LPA) and D-Dimer	Confirmed on Imaging

						volunteers were selected		
Sbarouni E(15)	2007	Greece	18	29 (21 dilated ascending aortas scheduled for surgery and 8 normal controls)	Consecutive AAD patients listed for surgery	Consective patients with chronic aortic aneurysm listed for surgery. Unclear how 8 normal subjects were selected	D-Dimer, White Blood Cell Count, C-Reactive Protein and Brain Natriuretic Protein	Confirmed on Imaging
Sbarouni E(16)	2018	Greece	120	242 (121 aortic aneurysm and 121 controls)	Consecutive AAD patients listed for surgery	Consecutive patients with aortic aneurysm listed for elective surgery. Unclear how controls were selected	Platelet to Lymphocyte Ratio, D-Dimer	Confirmed on Imaging
Sbarouni E(17)	2015	Greece	120 AAD	242 (121 Chronic Aneurysms and 121 healthy subjects)	Consecutive AAD patients listed for surgery	Consecutive patients with aortic aneurysm listed for elective surgery. Unclear how controls were selected	WBC Count, Neutrophil/Lymphocyte Ratio, D-Dimer and C-Reactive Protein	Unknown
Shinohara T(18)	2003	Japan	25	584 (50 AMI, 474 healthy controls, 20 other CP, 40 unmedicated hypertensive patients)	Unclear	Unclear	Soluble Elastin Fragments in Serum (s-ELAF)	Confirmed on Imaging

Xiao Z(19)	2016	China	60	60 CP with other diagnoses	Unclear	Unclear	Lumican, CRP, Thrombospondin-1 (TSP-1) and D-Dimer	Confirmed on Imaging
Xu Z(20)	2017	China	15 AAD + HTN	15 Controls with HTN	Unclear	Unclear	microRNAs	Confirmed on Imaging
Yuan SM(21)	2011	China	20	29 (9 Aortic Aneurysm and 20 CAD)	Consecutive AAD patients listed for surgery	Consective patients with aortic aneurysm listed for surgery. Unclear how CAD patients were selected	D-Dimer and hypersensitive CRP	Unclear
Zeng Q(22)	2020	China	19 AAD	20 Healthy Volunteers	Unclear	Unclear	Fasting Blood Glucose, Triglyceride, Low-Density Lipoprotein (LDL), D-Dimer, CRP, IL-6, Trimethylamine N-Oxide (TMAO), Carnitine, Choline and Betaine	Confirmed on Imaging
Cakir A(23)	2021	Turkey	20	20 Healthy Volunteers	Unclear	Unclear	Signal Peptide-CUB-EGF Domain-containing Protein-1 (SCUBE-1)	Confirmed on Imaging
Jiang Y(24)	2022	China	20	10 healthy volunteers	Unclear	Healthy controls gathered from the medical examination facility	Oxylipin profile	Confirmed on Imaging
Li T(25)	2022	China	25	15 healthy volunteers	Patients diagnosed with AAD within set temporal boundaries	Unclear	Genomescale leukocyte long noncoding RNAs (lncRNAs)	Confirmed on Imaging

Song R(26)	2022	China	57	57 acute coronary syndrome, 50 healthy volunteers	Patients diagnosed with AAD within set temporal boundaries	Unclear	Fibrinogen degradation products (FDP), fibrinogen (Fib), prothrombin time (PT), activated partial thromboplastin time (APTT), tenascin C (TN-C), D-dimer (D-D), and N-terminal Btype natriuretic peptide precursor (NT-proBNP)	Confirmed on Imaging
Zheng Z(27)	2012	China	118	94 chronic aneurism, 98 normal	Consecutive patients diagnosed with AAD within set temporal boundaries	Consecutive patients with aortic aneurysm listed for elective surgery. Unclear how controls were selected	D-dimer, CRP, BNP	Unclear
Xu Z(28)	2022	China	40 AAD	40 CP (17 STEMI, 11 unstable angina, 6 PE, 6 digestive tract disorder)	Patients diagnosed with AAD within set temporal boundaries	Patients presenting with CP within set temporal boundaries	D-Dimer, BNP and Troponin	Confirmed on Imaging
Zhao G(29)	2020	China	22 AAD	287 (268 MI, 19 PE)	Patients diagnosed with AAD within set temporal boundaries	Patients diagnosed with MI or PE within set temporal boundaries	D-Dimer	Confirmed on Imaging
Lu P(30)	2022	China	162 AD (Discovery Set); 105 AD (Validation Set)	168 (135 CAD, 33 other cardiovascular disease) [Discovery Set]; 86 non-AD [Validation Set]	Patients diagnosed with AAD within set temporal boundaries	Patients diagnosed with other cause of CP within set temporal boundaries	FLUTHE (Serum Iron, LDL, Uric Acid, Transferrin, High-Density Lipoprotein (HDL) and estimated Glomerular Filtration Rate (eGFR))	Confirmed on Imaging

Goliopoulou A(31)	2022	Greece	13 AAD	49 (24 aortic root dilatation, 25 other cardiac surgery)	Consecutive patients undergoing emergency surgical repair or Stanford Type A Dissection	Consecutive patients undergoing surgical repair of dilated aortic root or other cardiac surgery	High-Sensitivity CRP, Matrix Metalloproteinase-2 (MMP 2), MMP 9, osteoprotegerin, adiponectin, mRNA 29 and mRNA 195	Unclear
Suzuki T(32)	1996	Japan	27 AAD	65 MI	Patients admitted with AAD within set temporal boundaries	Unclear	Serum Smooth Muscle Myosin Heavy Chain	Confirmed on Imaging
Hazui H(33)	2005	Japan	29 AAD	49 MI	Consecutive patients diagnosed with AAD within set temporal boundaries	Consecutive patients diagnosed with MI within set temporal boundaries	D-Dimer, M-Ratio, Lactate Dehydrogenase, Myoglobin, Creatine Kinase, Haematocrit	Confirmed on Imaging
Hagiwara A(34)	2010	Japan	91 AAD	783 (190 Acute Myocardial Infarction, 142 Angina, 357 Brain Infarction, 94 Transient Ischaemic Attack)	Consecutive patients diagnosed with AAD within set temporal boundaries	Patient admitted within the same time period and subsequently diagnosed with MI, Angina, BI or TIA	Plasma Fibrin and Fibrin Degradation Products	Confirmed on Imaging
Suzuki T(35)	2011	Japan	28 AAD	Unclear	Unclear	Unclear	Transforming Growth Factor Beta	Unclear
Wagner A(36)	2002	Austria	18 AAD	47 (27 free or covered aneurysm	Consecutive patients diagnosed with	Consecutive patients presenting to the	Plasma Endothelin	Confirmed on Imaging

				rupture, 20 aortic aneurysm without signs of acute dissection or rupture)	AAD within set temporal boundaries	emergency department with known chronic aneurysm		
Fletcher A(37)	2021	United Kingdom	53 AAS	106 Controls	Patients over 25 years old with confirmed AAS	Control subjects participating in the United Kingdom Aneurysm Growth Study	Plasma Desmosine	Confirmed on Imaging
Zhang D(38)	2023	China	75 AAD	86 Controls	Patients confirmed as having AAD (no clear temporal boundaries)	Unclear	Exosomal miRNAs, Matrix Metalloproteinases 9 and 12, Transforming Growth Factor Beta and D-Dimer	Confirmed on Imaging

AAD (Acute Aortic Dissection); AD (Aortic Dissection); AAS (Acute Aortic Syndrome); CP (Chest Pain); PE (Pulmonary Embolism); MI (Myocardial Infarction); SPUM-ACS (Special Programme University Medicine – Acute Coronary Syndrome); SWITCO-65+ (Swiss Venous Thromboembolism Cohort 65+); CVA (Cerebrovascular Accident); TIA (Transient Ischaemic Attack); HTN (Hypertension); STEMI (ST-Elevation Myocardial Infarction); BI (Brain Infarction**)**

Appendix S3 References

1. Cheng N, Wang H, Zhang W, Wang H, Jin X, Ma X, et al. Comparative Proteomic Investigation of Plasma Reveals Novel Potential Biomarker Groups for Acute Aortic Dissection. Disease markers. 2020;2020:4785068.
2. Dong J, Bao J, Feng R, Zhao Z, Lu Q, Wang G, et al. Circulating microRNAs: a novel potential biomarker for diagnosing acute aortic dissection. Scientific reports. 2017;7(1):12784.
3. Dong J, Duan X, Feng R, Zhao Z, Feng X, Lu Q, et al. Diagnostic implication of fibrin degradation products and D-dimer in aortic dissection. Scientific reports. 2017;7:43957.
4. Eggebrecht H, Naber CK, Bruch C, Kroger K, von Birgelen C, Schmermund A, et al. Value of plasma fibrin D-dimers for detection of acute aortic dissection. Journal of the American College of Cardiology. 2004;44(4):804-9.
5. Forrer A, Schoenrath F, Torzewski M, Schmid J, Franke UFW, Gobel N, et al. Novel Blood Biomarkers for a Diagnostic Workup of Acute Aortic Dissection. Diagnostics (Basel, Switzerland). 2021;11(4).
6. Hagiwara A, Shimbo T, Kimira A, Sasaki R, Kobayashi K, Sato T. Using fibrin degradation products level to facilitate diagnostic evaluation of potential acute aortic dissection. Journal of thrombosis and thrombolysis. 2013;35(1):15-22.
7. Han C, Liu Q, Li Y, Zang W, Zhou J. S100A1 as a potential biomarker for the diagnosis of patients with acute aortic dissection. The Journal of international medical research. 2021;49(4):3000605211004512.
8. Konig C, Lahm H, DreBen M, Doppler S, Beck N, Holdenrieder S, et al. Aggrecan: A new biomarker for acute thoracic aortic dissection. Thoracic and Cardiovascular Surgeon Conference: 50th Annual Meeting of the German Society for Thoracic and Cardiovascular Surgery, DGTHG. 2021;69(SUPPL 1).
9. Li T, Jiang B, Li X, Sun H-Y, Li X-T, Jing J-J, et al. Serum matrix metalloproteinase-9 is a valuable biomarker for identification of abdominal and thoracic aortic aneurysm: a case-control study. BMC cardiovascular disorders. 2018;18(1):202.
10. Liu X, Zheng X, Su X, Tian W, Hu Y, Zhang Z. Plasma Resistin Levels in Patients with Acute Aortic Dissection: A Propensity Score-Matched Observational Case-Control Study. Medical science monitor : international medical journal of experimental and clinical research. 2018;24:6431-7.

11. Ohle R, Um J, Anjum O, Bleeker H, Luo L, Wells G, et al. High Risk Clinical Features for Acute Aortic Dissection: A Case-Control Study. Academic Emergency Medicine. 2018;25(4):378-87.
12. Ohlmann P, Faure A, Morel O, Petit H, Kabbaj H, Meyer N, et al. Diagnostic and prognostic value of circulating D-Dimers in patients with acute aortic dissection. Critical care medicine. 2006;34(5):1358-64.
13. Okazaki T, Yamamoto Y, Yoda K, Nagahiro S. The ratio of D-dimer to brain natriuretic peptide may help to differentiate between cerebral infarction with and without acute aortic dissection. Journal of the neurological sciences. 2014;340(1-2):133-8.
14. Pan X, Zhou Y, Yang G, He Z, Zhang H, Peng Z, et al. Lysophosphatidic Acid May Be a Novel Biomarker for Early Acute Aortic Dissection. Frontiers in surgery. 2021;8:789992.
15. Sbarouni E, Georgiadou P, Marathias A, Geroulanos S, Kremastinos DT. D-dimer and BNP levels in acute aortic dissection. International journal of cardiology. 2007;122(2):170-2.
16. Sbarouni E, Georgiadou P, Kosmas E, Analitis A, Voudris V. Platelet to lymphocyte ratio in acute aortic dissection. Journal of clinical laboratory analysis. 2018;32(7):e22447.
17. Sbarouni E, Georgiadou P, Analitis A, Voudris V. High neutrophil to lymphocyte ratio in type A acute aortic dissection facilitates diagnosis and predicts worse outcome. Expert review of molecular diagnostics. 2015;15(7):965-70.
18. Shinohara T, Suzuki K, Okada M, Shiigai M, Shimizu M, Maehara T, et al. Soluble elastin fragments in serum are elevated in acute aortic dissection. Arteriosclerosis, Thrombosis, and Vascular Biology. 2003;23(10):1839-44.
19. Xiao Z, Xue Y, Yao C, Gu G, Zhang Y, Zhang J, et al. Acute Aortic Dissection Biomarkers Identified Using Isobaric Tags for Relative and Absolute Quantitation. BioMed research international. 2016;2016:6421451.
20. Xu Z, Wang Q, Pan J, Sheng X, Hou D, Chong H, et al. Characterization of serum miRNAs as molecular biomarkers for acute Stanford type A aortic dissection diagnosis. Scientific reports. 2017;7(1):13659.
21. Yuan S-M, Shi Y-H, Wang J-J, Lu F-Q, Gao S. Elevated plasma D-dimer and hypersensitive C-reactive protein levels may indicate aortic disorders. Revista brasileira de cirurgia cardiovascular : orgao oficial da Sociedade Brasileira de Cirurgia Cardiovascular. 2011;26(4):573-81.

22. Zeng Q, Rong Y, Li D, Wu Z, He Y, Zhang H, et al. Identification of Serum Biomarker in Acute Aortic Dissection by Global and Targeted Metabolomics. Annals of vascular surgery. 2020;68:497-504.
23. Cakir A, Payza U, Aksun S, Kayali A, Karakaya Z, Topal FE. Validity of signal peptide-cub-egf domain-containing protein-1 (Scube-1) in the diagnosis of aortic dissection. Signa Vitae. 2021;17(1):112-6.
24. Jiang Y, Tang X, Wang Y, Chen W, Xue Y, Cao H, et al. Serum Oxylipin Profiles Identify Potential Biomarkers in Patients with Acute Aortic Dissection. Metabolites. 2022;12(7) (no pagination).
25. Li T, Zhou Y, Li D, Zeng Z, Zhang S. The role of genome-scale leukocyte long noncoding RNA in identifying acute aortic dissection. Signa Vitae. 2022;18(3):101-10.
26. Song R, Xu N, Luo L, Zhang T, Duan H. Diagnostic Value of Aortic Dissection Risk Score, Coagulation Function, and Laboratory Indexes in Acute Aortic Dissection. BioMed Research International. 2022;2022:7447230.
27. Ziyu Z, Zi Y, Jialin Y, Weiping W, Hong Z. Value of D-dimer for detection of acute aortic dissection. Heart. 2012;2):E268.
28. Xu Z, Wei M, Guo X, Zhang Q, Ma Y, Gao Z, et al. Changes of Serum D-Dimer, NT-proBNP, and Troponin I Levels in Patients with Acute Aortic Dissection and the Clinical Significance. Evidence-Based Complementary & Alternative Medicine: eCAM. 2022;2022:8309505.
29. Zhao G, Zhao Y, Zhang H. Value of duration of chest pain, troponin, and D-dimer in differentiating acute high-risk chest pain patient. Acta Medica Mediterranea. 2020;36(3):1587-91.
30. Lu P, Feng X, Li R, Deng P, Li S, Xiao J, et al. A Novel Serum Biomarker Model to Discriminate Aortic Dissection from Coronary Artery Disease. Disease Markers. 2022;2022:9716424.
31. Goliopoulou A, Oikonomou E, Antonopoulos A, Koumallos N, Gazouli M, Theofilis P, et al. Expression of Tissue microRNAs in Ascending Aortic Aneurysms and Dissections. Angiology. 2022:33197221098295.
32. Suzuki T, Katoh H, Watanabe M, Kurabayashi M, Hiramori K, Hori S, et al. Novel biochemical diagnostic method for aortic dissection: results of a prospective study using an immunoassay of smooth muscle myosin heavy chain. Circulation. 1996;93(6):1244-9.
33. Hazui H, Fukumoto H, Negoro N, Hoshiga M, Muraoka H, Nishimoto M, et al. Simple and useful tests for discriminating between acute aortic dissection of the ascending

aorta and acute myocardial infarction in the emergency setting. Circulation journal : official journal of the Japanese Circulation Society. 2005;69(6):677-82.

34. Hagiwara A, Sakamoto D, Sasaki R, Kobayashi K, Sato T, Kimira A. Diagnosis of acute aortic dissection using a fibrinolytic marker. Critical Care Medicine. 2010;12):A178.

35. Suzuki T, Trimarchi S, Sawaki D, Grassi V, Costa E, Rampoldi V, et al. Circulating transforming growth factor-beta levels in acute aortic dissection. J Am Coll Cardiol. 2011;58(7):775.

36. Wagner A, Domanovits H, Holzer M, Kofler J, Röggla M, Müllner M, et al. Plasma endothelin in patients with acute aortic disease. Resuscitation. 2002;53(1):71-6.

37. Fletcher A, Syed MBJ, Iskander Z, Debono S, Dweck MR, Huang J, et al. Plasma desmosine as a biomarker in acute aortic syndrome. European Heart Journal. 2021;42(1).

38. Zhang D, Zhao X, Wang B, Liu X, Aizezi A, Ma X. Circulating exosomal miRNAs as novel biomarkers for acute aortic dissection. Medicine. 2023;102(30):e34474.
