# Supplementary material for: Diagnostic accuracy of alternative biomarkers for acute aortic syndrome: a systematic review
Source: Emerg Med J. 2024 Aug 6;41(11):e213772. doi: 10.1136/emermed-2023-213772 (PMC11503200; doi:10.1136/emermed-2023-213772)
Supplement: online supplemental file 4 [file emermed-41-11-s004.pdf]

APPENDIX S4: QUADAS-2 quality assessment summary - Review authors' judgements in detail

	Chun & Siu, 2023(15)	Giachino et al. 2013(16)	Lian et al. 2023(17)	Meng et al. 2019(18)	Morello et al. 2016(19)	Morello et al. 2017(20)	Morello et al. 2018(21)	Morello et al. 2020(22)	Peng et al. 2015(23)	Suzuki et al. 2008(24)	von Kodolitsch et al. 2000(25)	Wang et al. 2018(26)	Zhang et al. 2023(27)
**Patient selection domain**													
1. Was a consecutive or random sample of patients enrolled?	No	Yes	Yes	No	No	Yes	No	Unclear	Unclear	No	Yes	Unclear	Yes
2. Was a case-control design avoided?	Yes	Yes	Yes	Yes	Yes	Yes	Yes	Yes	Yes	Yes	Yes	Yes	Yes
3. Did the study avoid inappropriate exclusions?	Unclear	Yes	Yes	No	Yes	Yes	Yes	Unclear	No	Unclear	Unclear	No	Yes
**SUMMARY: Risk of bias** Could the selection of patients have introduced bias?	**HIGH**	**LOW**	**LOW**	**HIGH**	**HIGH**	**LOW**	**HIGH**	**UNCLEAR**	**HIGH**	**HIGH**	**UNCLEAR**	**HIGH**	**LOW**
**SUMMARY: Concerns regarding applicability** Are there concerns that the included patients do not match the review question?	**LOW**	**LOW**	**LOW**	**HIGH**	**UNCLEAR**	**LOW**	**LOW**	**LOW**	**HIGH**	**UNCLEAR**	**UNCLEAR**	**HIGH**	**LOW**
**Index test domain**													
4. Were the index test results interpreted without knowledge of the results of the reference standard?	Unclear	Yes	Unclear	Unclear	Yes	Yes	Yes	Yes	Unclear	Unclear	Unclear	Unclear	Yes
5. If a threshold was used, was it pre-specified?	Yes	No	No	Yes	Yes	No	No	No	No	No	Unclear	No	No
**SUMMARY: Risk of bias** Could the conduct or interpretation of the index test have introduced bias?	**UNCLEAR**	**HIGH**	**HIGH**	**HIGH** [a]	**LOW**	**HIGH**	**HIGH**	**HIGH**	**HIGH**	**HIGH**	**UNCLEAR**	**HIGH**	**HIGH**
**SUMMARY: Concerns regarding applicability** Are there concerns that the index test, its conduct, or interpretation differ from the review question?	**LOW**	**LOW**	**LOW**	**HIGH** [a]	**LOW**	**LOW**	**LOW**	**LOW**	**LOW**	**LOW**	**LOW**	**LOW**	**LOW**
**Reference standard domain**													

6. Is the reference standard likely to correctly classify the target condition?	Yes	Yes	Yes	Yes	Yes	Yes	Yes	Yes	Yes	Unclear	Yes	Yes	Unclear
7. Were the reference standard results interpreted without knowledge of the results of the index test?	Unclear	Unclear	Unclear	Unclear	Yes	Yes	Yes	Yes	Unclear	Unclear	Unclear	Unclear	Yes
**SUMMARY: Risk of bias** Could the reference standard, its conduct, or its interpretation have introduced bias?	**UNCLEAR**	**UNCLEAR**	**UNCLEAR**	**UNCLEAR**	**LOW**	**LOW**	**LOW**	**LOW**	**UNCLEAR**	**UNCLEAR**	**UNCLEAR**	**UNCLEAR**	**UNCLEAR**
**SUMMARY: Concerns regarding applicability** Are there concerns that the target condition as defined by the reference standard does not match the review question?	**LOW**	**LOW**	**LOW**	**LOW**	**LOW**	**LOW**	**LOW**	**LOW**	**LOW**	**LOW**	**LOW**	**LOW**	**LOW**
**Flow and timing domain**													
8. Was there an appropriate interval between index test(s) and reference standard?	Unclear	Yes	Unclear	Unclear	Yes	Yes	Yes	Unclear	Yes	Yes	Unclear	Yes	Unclear
9. Did all patients receive a reference standard?	Unclear	Yes	Yes	Yes	Yes	Yes	Yes	Yes	Yes	Yes	Yes	Yes	Unclear
10. Did all patients receive the same reference standard?	Unclear	Yes	Yes	Yes	Yes	Yes	No	No	Yes	Unclear	No	Yes	No
11. Were all patients included in the analysis?	Yes	Unclear	Yes	Yes	Yes	Yes	Yes	Yes	Yes	Yes	Yes	Yes	Yes
**SUMMARY: Risk of bias** Could the patient flow have introduced bias?	**UNCLEAR**	**UNCLEAR**	**UNCLEAR**	**UNCLEAR**	**LOW**	**LOW**	**HIGH**	**HIGH**	**LOW**	**UNCLEAR**	**HIGH**	**LOW**	**HIGH**

[a] Although one domain was unclear; troponin test and cutoffs were changed during study period, thus assessed as high bias/applicability concerns

15. Chun H, Siu KM. A diagnostic and screening strategy with neutrophil counts in patients with suspected aortic dissection in a certain time window. World J Emerg Med. 2023;14(4):307-11.

16. Giachino F, Loiacono M, Lucchiari M, Manzo M, Battista S, Saglio E, et al. Rule out of acute aortic dissection with plasma matrix metalloproteinase 8 in the emergency department. Critical care (London, England). 2013;17(1):R33.
17. Lian R, Zhang T, Liu J, Zhang G, Hu T, Li G, et al. Routine Use of a Pocket-Sized Handheld Echoscopic Device

Plus a Biomarker by Emergency Medicine Residents with an

Early Screening Algorithm for Suspected Type A Acute

Aortic Syndrome. Journal of Clinical Medicine. 2023;12:1346.
18. Meng J, Mellnick VM, Monteiro S, Patlas MN. Acute Aortic Syndrome: Yield of Computed Tomography Angiography in Patients With Acute Chest Pain. Canadian Association of Radiologists Journal. 2019;70(1):23-8.
19. Morello F, Ravetti A, Nazerian P, Liedl G, Veglio MG, Battista S, et al. Plasma Lactate Dehydrogenase Levels Predict Mortality in Acute Aortic Syndromes: A Diagnostic Accuracy and Observational Outcome Study. Medicine. 2016;95(6):e2776.
20. Morello F, Cavalot G, Giachino F, Tizzani M, Nazerian P, Carbone F, et al. White blood cell and platelet count as adjuncts to standard clinical evaluation for risk assessment in patients at low probability of acute aortic syndrome. European heart journal Acute cardiovascular care. 2017;6(5):389-95.
21. Morello F, Oddi M, Cavalot G, Ianniello A, Giachino F, Nazerian P, et al. Prospective diagnostic and prognostic study of copeptin in suspected acute aortic syndromes. Scientific reports. 2018;8(1):16713.
22. Morello F, Bartalucci A, Bironzo M, Santoro M, Pivetta E, Ianniello A, et al. Prospective diagnostic accuracy study of plasma soluble ST2 for diagnosis of acute aortic syndromes. Scientific reports. 2020;10(1):3103.
23. Peng W, Peng Z, Chai X, Zhu Q, Yang G, Zhao Q, et al. Potential biomarkers for early diagnosis of acute aortic dissection. Heart & lung : the journal of critical care. 2015;44(3):205-8.
24. Suzuki T, Distante A, Zizza A, Trimarchi S, Villani M, Salerno Uriarte JA, et al. Preliminary experience with the smooth muscle troponin-like protein, calponin, as a novel biomarker for diagnosing acute aortic dissection. European heart journal. 2008;29(11):1439-45.
25. von Kodolitsch Y, Schwartz AG, Nienaber CA. Clinical prediction of acute aortic dissection. Archives of internal medicine. 2000;160(19):2977-82.
26. Wang Y, Tan X, Gao H, Yuan H, Hu R, Jia L, et al. Magnitude of Soluble ST2 as a Novel Biomarker for Acute Aortic Dissection. Circulation. 2018;137(3):259-69.
27. Zhang H, Yuan N, Guo J, Hou M. Comparisons of potential values of D-dimer and the neutrophil-to-lymphocyte ratio in patients with suspected acute aortic syndrome. The American Journal of Emergency Medicine. 2023;69:44-51.
