# Supplementary material for: Diagnostic accuracy of alternative biomarkers for acute aortic syndrome: a systematic review
Source: Emerg Med J. 2024 Aug 6;41(11):e213772. doi: 10.1136/emermed-2023-213772 (PMC11503200; doi:10.1136/emermed-2023-213772)
Supplement: online supplemental file 5 [file emermed-41-11-s005.docx]

**Appendix S5: Summary of accuracy results (sensitivity, specificity, positive and negative predictive values, positive and negative likelihood ratios and AUROC) along with their respective 95% confidence intervals and cut-off values where provided for the biomarkers investigated in the 13 included cohort studies**

| **Author, year** | **Biomarker** | **Sensitivity (%)** | **95% CI** | **Specificity (%)** | **95% CI** | **PPV (%)** | **NPV (%)** | **LR+** | **LR-** | **AUROC** | **95% CI** | **Cut-Off ^a^** |
| --- | --- | --- | --- | --- | --- | --- | --- | --- | --- | --- | --- | --- |
| Chun et al. 2023(14) | Neutrophil Count (2-8 hours after symptom onset) | 94.8 | 84.7, 98.6 | 59.4 | 50, 68.4 | 53.9 | 95.8 | 2.34 | 0.09 | NR | NR | ≥6.2 x 10^9^/L |
|  | Neutrophil Count (8-24 hours) | 96.9 | 82, 99.8 | 45 | 29.6, 61.3 | 58.5 | 94.7 | 1.76 | 0.07 | NR | NR |  |
|  | Neutrophil Count (2-24 hours) | 95.6 | 88.4, 98.6 | 56.1 | 47.9, 63.9 | 55.5 | 98.6 | 2.71 | 0.08 | NR | NR |  |
|  | Neutrophil Count (2-24 hours) and ADD Risk Score ≤1) | 94.6 | 84.2, 98.6 | 52.3 | 42.1, 61.9 | NR | NR | NR | NR | NR | NR |  |
| Giachino et al. 2013(15) | MMP8 | 100 | 93.2, 100 | 9.5 | 3.9, 18.5 | NR | 84.2 (With cut-off 11ng/mL) | NR | NR | 0.75 | NR | 3.6 ng/mL |
|  | MMP9 | 96.2 | 86.8, 99.5 | 16.2 | 8.7, 26.6 | NR | NR | NR | NR | 0.7 | NR | 20 ng/mL |
|  | D-Dimer | 97.6 | 87.4, 99.9 | 32.8 | 21.3, 46 | NR | NR | NR | NR | 0.87 | 0.8, 0.94 | 500 ng/mL |
|  | Log2 D-dimer and MMP8 | 100 | 91.6, 100 | 13.1 | 5.8, 24.2 | NR | 100 (With MMP8 cut-off 11ng/mL and any D-dimer cut-off) | NR | NR | 0.89 | 0.82, 0.95 | >0.77 |
| Lian et al. 2023(16) | Acidic Calponin | 77.6 | NR | 87.7 | NR | NR | NR | NR | NR | 0.889 | NR | 6.96 ng/mL |
|  | Acidic Calponin + Ascending Aortic Root Dilation | 83.7 | NR | 89.2 | NR | NR | NR | NR | NR | 0.927 | NR | Calponin 6.96 ng/mL and diameter >40mm |
| Meng et al. 2019(17) | Troponin | 16.7 | NR | 76.7 | NR | NR | NR | NR | NR | NR | NR | >0.04 μg/mL (old), ≥30 μg/L (new) |
|  | D-Dimer | 100 | NR | 51.3 | NR | NR | NR | NR | NR | NR | NR | >500 ng/mL |
| Morello et al. 2016(18) | LDH | 44 | 37, 51 | 73 | 69, 76 | 29 | 84 | 1.61 | 0.77 | 0.61 | 0.57, 0.66 | 450 U/L |
| Morello et al. 2017(19) | WBC Count | 67.3 | 57.7, 75.9 | 59 | 55.5, 62.5 | NR | NR | NR | NR | 0.69 | 0.63, 0.74 | >9*10^3^/μL |
|  | Platelet Count | 68.2 | 58.6, 76.7 | 56.2 | 52.7, 59.7 | NR | NR | NR | NR | 0.64 | 0.58, 0.69 | >200*10^3^/μL |
|  | Fibrinogen | 50.9 | 41.2, 60.2 | 63.6 | 60.2, 67 | NR | NR | NR | NR | 0.62 | 0.55, 0.68 | <350 mg/dL |
|  | ≥ 1 alteration(s) | 95.5 | 89.7, 98.5 | 18.3 | 15.7, 21.2 | NR | NR | NR | NR | NR | NR | N/A |
| Morello et al. 2018(20) | Copeptin | 78.8 | 70.1, 85.6 | 74.6 | 68.3, 80.1 | 60.7 | 87.6 | 3.11 | 0.28 | 0.81 (AAS), 0.83 (AAD] | 0.75, 0.86 (AAS), 0.77, 0.88 (AAD) | 14 pmol/L |
|  | D-Dimer | 95.2 | 88.3, 98.1 | 65.4 | 58.5, 71.8 | NR | NR | NR | NR | 0.92 | 0.89, 0.96 | ≥500 ng/mL |
|  | Copeptin + D-dimer | 95.2 | 88.3, 98.1 | 46.6 | 39.7, 53.7 | NR | NR | NR | NR | 0.92 | 0.88, 0.95 | D-dimer <500 ng/mL and copeptin <10 pmol/L |
| Morello et al. 2020(21) | sST2 | 58 | 47, 68.4 | 70.8 | 64.1, 76.9 | NR | NR | 1.99 | 0.59 | 0.675 | 0.61, 0.736 | 39.8 ng/mL |
|  |  |  |  |  |  |  |  |  |  | 0.717 (when low pre-test risk) | 0.655, 0.772 |  |
|  | D-Dimer | 95.8 | 88.1, 99.1 | 30.7 | 19.6, 43.7 | NR | NR | 1.38 | 0.14 | 0.842 | 0.753, 0.908 | 500 ng/mL |
| Peng et al. 2015(22) | Alpha-SMA | 54.29 | NR | 90.24 | NR | NR | NR | NR | NR | 0.62 | 0.49, 0.76 | 49.62 ng/mL |
|  | smMHC | 68.57 | NR | 90.24 | NR | NR | NR | NR | NR | 0.81 | 0.71, 0.91 | 2.11 ng/mL |
|  | sELAF | 82.86 | NR | 68.29 | NR | NR | NR | NR | NR | 0.82 | 0.73, 0.91 | 97.07 ng/mL |
|  | PC1 | 85.71 | NR | 75.61 | NR | NR | NR | NR | NR | 0.9 | 0.83, 0.96 | 357.33 pg/mL |
|  | 1 Variable Positive | 100 | NR | 53.66 | NR | NR | NR | NR | NR | NR | NR | N/A |
|  | 2 Variables Positive | 94.29 | NR | 85.37 | NR | NR | NR | NR | NR | 0.95 | 0.87, 0.99 | N/A |
|  | 3 Variables Positive | 77.14 | NR | 95.12 | NR | NR | NR | NR | NR | NR | NR | N/A |
|  | All Positive | 51.43 | NR | 100 | NR | NR | NR | NR | NR | NR | NR | N/A |
|  | D-Dimer | 80 | NR | 90.21 | NR | NR | NR | NR | NR | 0.93 | 0.87, 0.98 | >2110 ng/mL |
| Suzuki et al. 2008(23) | Acidic Calponin (Initial 6h) | 50 | NR | 87 | NR | 56 | 84 | NR | NR | 0.63 | NR | 2.8 ng/L |
|  | Acidic Calponin (Initial 24h) | 58 | NR | 72 | NR | 41 | 84 | NR | NR | 0.63 | NR | 2.3 ng/L |
|  | Basic Calponin (Initial 6h) | 63 | NR | 73 | NR | 44 | 86 | NR | NR | 0.67 | NR | 159 ng/L |
|  | Basic Calponin (Initial 24h) | 50 | NR | 66 | NR | 33 | 80 | NR | NR | 0.58 | NR | 139 ng/L |
| von Kodolitsch et al. 2000(24) | Leukocyte Count | 25.8 | NR | 77.9 | NR | NR | NR | NR | NR | NR | NR | ≥15 x 10^9^/L |
| Wang et al. 2018(25) | Soluble ST2 | 99.1 | NR | 84.9 | NR | 68.7 | 99.7 | 6.6 | 0.01 | 0.97 | 0.95, 0.98 | 34.6 ng/mL |
|  | Troponin | NR | NR | NR | NR | NR | NR | NR | NR | 0.5 | 0.44, 0.56 | NR |
|  | D-Dimer | 93.9 | NR | 78.5 | NR | NR | NR | NR | NR | 0.91 | 0.88, 0.94 | 323 ng/mL |
| Zhang et al. 2023(26) | Neutrophil-Lymphocyte Ratio | 76 | 71, 81 | 79 | 74, 83 | 54 | 91 | 3.6 | 0.3 | 0.845 | 0.816, 0.871 | NR |
|  | D-Dimer | 74 | 69, 79 | 76 | 72, 80 | 51 | 90 | 3.1 | 0.34 | 0.822 | 0.792, 0.850 | NR |

MMP (Matrix Metalloproteinase); LDH (Lactate Dehydrogenase); WBC (White Blood Cell); Alpha-SMA (Alpha-Smooth Muscle Actin); smMHC (Smooth Muscle Myosin Heavy Chain); sELAF (Soluble Elastin Fragments); PC1 (Polycystin-1); sST2 (Soluble Suppression of Tumourigenicity-2); ng/mL (nanogram per millilitre); U/L (Units per litre); μL (microlitre); mg/dL (milligram per decilitre); μg/mL (microgram per millilitre); μg/L (microgram per litre); ng/mL (nanogram per millilitre); pmol/L (picomole per litre); ng/L (nanogram per litre); L (litre); ADD (Aortic Dissection Detection); mm (millimetres); NR (Not Reported)

**^a^** Morello 2016(18) and Chun 2023(14) used the standard laboratory cut-offs; Giachino 2013(15), Morello 2018(20), Morello 2020(21), Peng 2015(22), Wang 2018(25) and Lian 2023(16) all used accuracy data to determine the optimal cut-off and von Kodolitsch 2000(24) and Zhang 2023(26) did not record how the cut-off was reached.
